# Gibt es einen fachspezifischen Medieneinsatz im naturwissenschaftlichen Fachunterricht? Ergebnisse einer Fragebogenerhebung

**DOI:** 10.1007/s40573-021-00130-5

**Published:** 2021-06-18

**Authors:** Hendrik Härtig, Anje Ostermann, Mathias Ropohl, Julia Schwanewedel, Lorenz Kampschulte, Anke Lindmeier

**Affiliations:** 1 Universität Duisburg-Essen, Universitätsstraße 2, 45127 Essen, Deutschland; 2 Leibniz-Institut für die Pädagogik der Naturwissenschaften und Mathematik, Olshausenstraße 62, 24118 Kiel, Deutschland; 3 Universität Hamburg, Alsterterrasse 1, 20354 Hamburg, Deutschland; 4 Deutsches Museum, Museumsinsel 1, 80538 München, Deutschland; 5 Friedrich-Schiller-Universität Jena, Ernst-Abbe-Platz 2, 07743 Jena, Deutschland

**Keywords:** Lehrkräfte, Einstellungen, Medieneinsatz, Science teachers, Attitudes, Use of media

## Abstract

Mediennutzung wird bislang selten fachspezifisch beschrieben. Der Fokus liegt meist entweder auf der Ausstattung und Nutzung im Allgemeinen – und damit weniger auf der Situation in einzelnen Fächern – oder auf der fachspezifischen Beschreibung für nur einzelne Medien. Aus naturwissenschaftsdidaktischer Perspektive ist aber gerade die fachspezifische Funktion des Medieneinsatzes im Lehr-Lern-Prozess bedeutsam. Dies legt eine theoretisch begründete Heuristik der Mediennutzung nahe, die unter anderem die fachdidaktische Tiefenstruktur der Mediennutzung in den Blick nimmt. In diesem Beitrag werden erste Indizien für fachdidaktische Potenziale von Medien mit Blick auf Lernprozesse in den naturwissenschaftlichen Fächern vorgestellt. Im Vergleich verschiedener exemplarischer Medien zwischen den drei Fächern Biologie, Chemie und Physik werden damit frühere Untersuchungen, z. B. zu einzelnen Medien wie dem Computer erweitert. Untersucht werden die Verfügbarkeit und Nutzung insbesondere gegenständlicher Modelle sowie digitaler Medien. Es ergeben sich in bestimmten Unterrichtssituationen Fachspezifika, die als Beleg für die vorgeschlagene Heuristik interpretiert werden können.

**Zusatzmaterial online:**

Zusätzliche Informationen sind in der Online-Version dieses Artikels (10.1007/s40573-021-00130-5) enthalten.

## Abstract (en)

Media use has so far hardly been described in a subject-specific manner. In previous studies, the focus was either on equipment and use in general and thus less on the situation in individual subjects; or on subject-specific descriptions for only single media. From a science education perspective, however, the subject-specific function of media for teaching and learning is significant. This deficit takes up a heuristic of media use, which among other things considers the deep structure of media use. In this article first indications for such didactic potential of media are presented. By comparing different exemplary media between biology, chemistry, and physics, earlier results, e.g for hardware, are extended. The availability and use of models and digital media are examined. Subject specifics arise in certain teaching situations that can be interpreted as evidence for the proposed heuristic.

## Einleitung

Bildung in der digitalen Welt erfordert die Entwicklung fachdidaktisch begründeter Konzepte unter Einbezug neuer Medien im Sinne einer Unterrichtsentwicklung (KMK 2016). In diesem Zuge ist inzwischen häufig untersucht worden, mit welchen Medien eine Schule ausgestattet wird und welche der Medien Lehrkräfte als zentrale Akteure der Unterrichtsplanung und -durchführung nutzen. Nicht zuletzt durch die Studie ICILS 2013 ist die Ausstattung von Schulen mit digitalen Medien und deren Nutzung durch Lehrkräfte auch in den Fokus der Bildungsforschung gerückt (Bos et al. 2014). Dabei hat sich die Forschung allerdings stark auf die Ausstattung von Schulen sowie auf die Nutzung der verfügbaren digitalen Medien durch Lehrkräfte im Allgemeinen gerichtet und weniger die Situation in einzelnen Fächern, Klassen oder Fächergruppen analysiert (Eickelmann et al. 2017; Lorenz et al. 2017b). Aus einer naturwissenschaftsdidaktischen Perspektive interessiert neben der bloßen Verfügbarkeit und Nutzungshäufigkeit vor allem das fachdidaktische Potenzial bestimmter Medien im fachlichen Lehr-Lern-Prozess: Wofür wird ein Medium – sei es ein analoges oder digitales – konkret im naturwissenschaftlichen Unterricht eingesetzt? Diese Frage ist bislang nicht im konkreten Fachvergleich und/oder Medienvergleich untersucht worden, sondern nur mit Blick auf einzelne Medien in einzelnen Fächern (z. B. Pietzner 2009). Dieser Beitrag stellt eine Untersuchung vor, die die Mediennutzung praktizierender Lehrkräfte der Naturwissenschaften mit Blick auf den Fachunterricht in Physik, Chemie und Biologie untersucht. Dabei werden exemplarisch analoge und digitale, traditionelle und moderne Medien analysiert. Ziel der Untersuchung war vor allem der Vergleich zwischen den drei Fächern anhand einer Auswahl von Medien, um erste Indizien zu sammeln, inwieweit Lehrkräfte Funktionen innerhalb der Naturwissenschaften (fachspezifisch) erkennen bzw. nutzen.

## Fachdidaktische Potenziale von Medien im naturwissenschaftlichen Unterricht

Medien werden im Unterricht seit jeher als Mittler zwischen Lehrenden, Lernenden und Lerninhalt eingesetzt. Diese Beziehungen – repräsentiert im sogenannten didaktischen Dreieck – greift die Definition der Mediendidaktik zum Medienbegriff auf, nach der Medien sowohl eine kognitive als auch eine kommunikative Funktion haben (Petko 2014). Mit Blick auf den konkreten Medieneinsatz bedeutet dies, dass Medien zur Prüfung und Beurteilung, zur Lernberatung und Kommunikation, zur Gestaltung von Lernaufgaben, als Werkzeug und Arbeitsmittel sowie als Informations- und Präsentationsmittel genutzt werden können. Aus Sicht der Naturwissenschaftsdidaktiken interessiert insbesondere die kognitive Funktion von Medien als Werkzeug und Arbeitsmittel, da hier starke Bezüge zu fachimmanenten und domänenspezifischen Anforderungen an das Lehren und Lernen gesehen werden können, beispielsweise im Zusammenhang mit dem Experimentieren oder dem Modellieren (Nerdel 2017; Reiners 2017; Steffensky et al. 2018). Ziel des Medieneinsatzes sind die Nutzung oder Erstellung externer Repräsentationen, die den Lernenden den Aufbau kohärenter Wissensstrukturen und den Erwerb anschlussfähiger Kompetenzen ermöglichen.

Dieses Ziel begründet sich durch die spezifische Herausforderung des naturwissenschaftlichen Unterrichts, mit den Sinnen erfahrbare, naturwissenschaftliche Phänomene mit eher kognitiven, abstrakten Konzepten in Verbindung zu setzen (Sumfleth und Nakoinz 2019). Um diese Verbindung fachdidaktisch anzubahnen, werden Medien auf der Seite der Phänomene (z. B. Schrader und Schanze 2015; Sieve et al. 2015) gezielt mit der Seite der Konzepte (z.  B. Lindenstruth et al. 2019) verknüpft. So dienen die Medien als Mittler zwischen diesen beiden Seiten im Sinne multipler externer Repräsentationen und erfüllen damit eine (nicht beliebige) fachdidaktische Funktion (Gilbert und Treagust 2009).

Aus Sicht des jeweiligen Fachs ist im Hinblick auf die Unterrichtsqualität ein möglichst spezifischer Medieneinsatz als Teil des Lehr-Lern-Prozesses von Bedeutung (McKnight et al. 2016). Doch selbst fach- oder domänenspezifische Angebots-Nutzungs-Modelle weisen Medien und ihren Einsatz nicht als explizite Merkmale aus (Neuhaus 2007), gleichwohl fachspezifische Merkmale wie die Fokussierung auf wesentliche naturwissenschaftliche Fachbegriffe, die Einbettung von naturwissenschaftlichen Experimenten oder die Nutzung von naturwissenschaftlichen Modellen nicht ohne den Einsatz von Medien umgesetzt werden können. Beispielsweise ist bei Steffensky und Neuhaus (2018) die Nutzung geeigneter Medien ein Indikator für Unterrichtsstrukturierung, detaillierte Angaben zur Ausgestaltung des Medieneinsatzes finden sich jedoch nicht.

Insgesamt erscheint es offensichtlich, dass Medien im naturwissenschaftlichen Unterricht in ihrer kognitiven Funktion bedeutsam sind. Andererseits fehlt es an Arbeiten, in denen jenseits allgemeiner Zuschreibungen spezifische fachdidaktische Potenziale bestimmten Medien zur Erfüllung dieser Funktion zugeschrieben oder untersucht werden. Bei der Untersuchung von Pietzner (2009) wird dies in Teilen für Computer und Software geleistet, wenn dort in der Gruppe der „Nutzer“ zum Beispiel Messwerterfassung oder Moleküldarstellungen sogar als fachspezifische Einsätze herausgestellt werden. Einerseits fehlt dort aber ein Vergleich zu analogen Medien, andererseits fehlt – vor dem Hintergrund des Zeitpunkts der Untersuchung – eine Anbindung an den Kompetenzbegriff.

Um insbesondere dem ersten Defizit zu begegnen, hat das Autorenteam eine Heuristik zur Beschreibung und Analyse der fachdidaktischen Potenziale des Medieneinsatzes vorgeschlagen. Ziel ist eine stärkere Systematisierung der Beschreibung des Potenzials spezifischer Medien, unabhängig von sonstigen Klassifikationen wie analog oder digital, traditionell oder modern, mit Blick auf die Unterrichtsqualität sowie die konkrete Verknüpfung des Medieneinsatzes mit dem Lehr-Lern-Prozess und den damit verbundenen Zielen (Härtig et al. 2018; Ropohl et al. 2018). Die erste Ausgangsüberlegung zu der Heuristik ist die Unterscheidung zwischen dem Medium selbst auf der einen Seite und dem Medieneinsatz auf der anderen Seite. Damit wird zwischen dem, was ein Medium „kann“ und dem, was davon in einer konkreten Situation „genutzt wird“, differenziert. Die zweite Ausgangsüberlegung ist – in Anlehnung an die Unterrichtsqualitätsforschung – die Unterscheidung zwischen der Oberflächenstruktur und der Tiefenstruktur von Unterricht (Oser und Baeriswyl 2002; Decristan et al. 2020). Aus diesen beiden Überlegungen ergibt sich eine Heuristik, mit der sowohl das Medium als auch der Medieneinsatz sowohl auf der Oberflächen- als auch auf der Tiefenstrukturebene betrachtet werden können im Hinblick auf (1) Eigenschaften des Mediums bezüglich der Oberflächenstruktur von Unterricht, (2) Eigenschaften des Mediums bezüglich der Tiefenstruktur von Unterricht, (3) Eigenschaften des Medieneinsatzes bezüglich der Oberflächenstruktur von Unterricht und (4) Eigenschaften des Medieneinsatzes bezüglich der Tiefenstruktur von Unterricht (s. Ropohl et al. 2018). Mithilfe dieser Heuristik können bestimmte Medieneinsätze im naturwissenschaftlichen Fachunterricht differenziert beschrieben werden, wobei besonders der vierte Punkt für die Bestimmung des Potenzials mit Blick auf fachliche Lernprozesse ausschlaggebend scheint. Zunächst ist aber zu prüfen, inwieweit die jeweiligen, für das Fach typischen Medien als notwendige Bedingung verfügbar sind und ob die im Rahmen der Heuristik angenommenen Potenziale einer Nutzung (fachspezifisch) ausgeschöpft werden.

## Befunde zur Verfügbarkeit und zur Nutzungshäufigkeit von Medien im naturwissenschaftlichen Unterricht

In den letzten Jahren sind eine Reihe von Untersuchungen und Befragungen von unterschiedlichen Akteuren durchgeführt worden, die zu verschiedenen Zeitpunkten die Ausstattung von Schulen mit Medien insgesamt sowie deren Nutzung durch Lehrkräfte erhoben haben. Bis dato liefern die Daten der Umfragen ein zweigeteiltes Bild. Die Umfrageergebnisse belegen, dass ein Großteil der Schulen der befragten Lehrkräfte mit Beamern, Notebooks, Kameras, Desktop Computern, DVD-/Blue-ray-Playern und Whiteboards ausgestattet sind (BITKOM e. V. 2015; Forsa 2014; Initiative D21 e. V. 2016). Seltener waren Tablet-Computer vorhanden. Die Befunde des Länderindikators 2017 (Lorenz und Endberg 2017) belegen zudem, dass es innerhalb Deutschlands zwischen den Bundesländern starke regionale Unterschiede hinsichtlich der Medienausstattung gibt. In den Bundesländern Schleswig-Holstein und Nordrhein-Westfalen liegt die Ausstattung der Schulen mit Medien beispielsweise auf einem mittleren Niveau im Vergleich zu der Ausstattung in allen anderen Bundesländern. Gleichzeitig legen die Antworten der Lehrkräfte offen, dass die Mehrzahl digitale Medien wie Whiteboards, Beamer, Tablets, Laptops oder Computer im Unterricht (über alle Klassen hinweg) nur gelegentlich einsetzt und nur etwa ein Drittel häufig (Forsa 2014). Auf internationaler Ebene wurden im Rahmen der Vergleichsstudien ICILS 2013 und ICILS 2018 (Bos et al. 2014; Eickelmann et al. 2019) deutsche Lehrkräfte zur Nutzungshäufigkeit digitaler Medien für schulische Zwecke befragt. In der Studie von 2013 gaben 9,1 % der befragten Lehrkräfte an, täglich digitale Medien zu nutzen, 34,4 % nutzten sie laut der Befragung mindestens wöchentlich. Damit lag die Gruppe der deutschen Lehrkräfte im internationalen Vergleich von ICILS 2013 auf dem letzten Platz des Rankings (Eickelmann et al. 2014), obwohl auch häusliche Recherche- oder Vorbereitungsarbeiten hier eingehen. Auch in der Befragung im Rahmen von ICILS 2018 landet die Gruppe der befragten deutschen Lehrkräfte auf den hinteren Plätzen, obwohl nun 23,1 % der Lehrkräfte angaben, digitale Medien für schulische Zwecke täglich zu nutzen und 60,2 % wöchentlich (Drossel et al. 2019). Dies führt zu der Vermutung, dass die bloße Verfügbarkeit nicht zwingend zur Nutzung führt.

Die berichteten sehr allgemeinen Befunde lassen sich im Rahmen einer Befragung von MINT-Lehrkräften für den Länderindikator 2017 anhand von fünf Einsatzszenarien spezifizieren. Definiert wurden: „(1) die Arbeit mit Textverarbeitungsprogrammen, (2) die Gestaltung von Präsentationen, (3) die Nutzung von Anwendungen zur Datenerfassung und -bearbeitung, (4) die Arbeit mit Tabellenkalkulationsprogrammen sowie (5) die Arbeit mit Simulations‑, Experimentier- oder Modellbildungs‑/Modellierungsprogrammen“ (Eickelmann et al. 2017, S. 240). Damit steht die fachbezogene Nutzung der Medien im Unterricht im Vordergrund, wobei wie in den anderen Studien die Analyseeinheit Lehrkraft gewählt wurde, was die Nutzungsangaben mit Blick auf einzelne Klassen überschätzt. Für die fünf genannten Aktivitäten geben zwischen 20 und 29 % der befragten Lehrkräfte an, dass diese regelmäßig – das bedeutet in dieser Befragung mindestens einmal wöchentlich – mit digitalen Medien durchgeführt werden (Eickelmann et al. 2017). Domänenspezifische Unterschiede werden für die beiden Aktivitäten Arbeit mit Tabellenkalkulationsprogrammen und Arbeit mit Simulations‑, Experimentier- oder Modellbildungs‑/Modellierungsprogrammen berichtet. Für diese Aktivitäten werden von den MINT-Lehrkräften (27,2 % für die Arbeit mit Tabellenkalkulationsprogrammen und 24,4 % für die Arbeit mit Simulations‑, Experimentier- oder Modellbildungs‑/Modellierungsprogrammen) signifikant häufiger digitale Medien genutzt als von Nicht-MINT-Lehrkräften (17,8 % bzw. 17,7 %). Da beide Aktivitäten domänenspezifische Arbeitsweisen darstellen, ist dies erwartungskonform. Durch diese Befunde wird einerseits deutlich, dass der MINT-Unterricht spezifische Funktionen der Medien aufgreift, andererseits ist unklar, ob dies für alle Fächer gleichermaßen gilt, da hier nur MINT- versus Nicht-MINT-Fächer verglichen werden. Ferner beschränkt sich die Erhebung auf digitale Medien und lässt tradierte Medien wie gegenständliche Modelle außen vor.

In einer Videostudie wurden Einsatzszenarien von Medien im Biologieunterricht untersucht (Kramer et al. 2019). Basis ist eine Beschreibung von zehn möglichen, sowohl kommunikativen wie kognitiven Funktionen von Medien am Beispiel des Computers (Schaal et al. 2013): (1) Recherche und Information, (2) Übung, (3) Messen und Rechnen, (4) Kommunikation und Kollaboration, (5) Exploration, (6) Visualisierung, (7) Präsentation, (8) Produktion und Dokumentation, (9) Strukturierung, (10) On-Modus und (11) Sonstiges. Mittels *N* = 85 Unterrichtsvideos von *N* = 43 bayrischen Gymnasiallehrkräften wurden die darin zu beobachtenden Medien mit ihrer jeweiligen Funktion codiert. Für das Fach Biologie sind die Präsentation sowie Produktion und Dokumentation gemessen an der Anzahl der Unterrichtsstunden, in denen sie zu beobachten sind, von hoher Bedeutung (Kramer et al. 2019). Alle anderen Funktionen von Medien konnten gar nicht oder nur sehr selten beobachtet werden. Auf diese Weise konnte für den Biologieunterricht beispielhaft die Verteilung der Mediennutzung auf Unterrichtsstunden beschrieben werden. Dies stellt eine höhere Auflösung als die oben berichteten Angaben auf Lehrkräfteebene dar. Gleichzeitig wurden die Lehrkräfte nicht spezifisch befragt, mit welchem (fachdidaktischen) Ziel der Einsatz eines Mediums verbunden war. So ließe sich zwar eventuell über eine Korrelation aus Funktion und Medium auf das Potenzial schließen, dies bleibt aber hypothetisch. Ebenfalls bezogen auf Computer untersucht Pietzner (2009) im Fächervergleich Nutzungshäufigkeit und -zweck. In den Ergebnissen wird zwischen „Meidern“ und „Nutzern“ unterschieden. Die „Meider“ werden hinsichtlich ihrer Begründungen vertieft betrachtet, die „Nutzer“ hinsichtlich der Einsatzszenarien. Auch in dieser Studie zeigen sich – eben für den Computereinsatz – die hier vorgeschlagenen Fachspezifika. Während der Einsatz mancher Funktionen, wie E‑Mail oder Chat, wenig fachdidaktisch begründbar erscheint, werden aber auch spezifische Funktionen, wie Messwerterfassung oder Animationen in den Blick genommen. Dabei zeigen sich tatsächlich differentielle Befunde.

Insgesamt deutet sich damit an, dass die Verfügbarkeit insbesondere digitaler Medien im Allgemeinen untersucht ist, eine fachspezifische Nutzungshäufigkeit verschiedener Medien (zum Beispiel auch das Schulbuch oder gegenständliche Modelle) jedoch nicht. Zu fachdidaktischen Potenzialen einzelner Medien aus Sicht der Lehrkraft ist ebenfalls wenig bekannt. Da die Nutzungsangaben sich meist auf die Lehrkraftebene beziehen und teilweise außerunterrichtliche schulische Nutzungen umfassen, lassen sich nur vage Schlüsse auf die Nutzung im Unterricht einzelner Klassen ziehen. Hinsichtlich der Intention ist unklar, ob es hier schlicht um Medienvielfalt geht oder ob Lehrkräfte dezidierte Potenziale bestimmter Medien für bestimmte Lerninhalte mitdenken. Hier zeigen Untersuchungen und Modellrechnungen (Mayer und Girwidz 2019), dass Lehrkräfte in der Tat einen Bedarf an fachspezifischen Informationen zum Medieneinsatz haben. Allerdings sind die Einflüsse auf die tatsächliche Mediennutzung im Unterricht mehrschichtig und komplex, wie in dem untersuchten Akzeptanzmodell deutlich wird (Mayer und Girwidz 2019). Neben der reinen Verfügbarkeit wird auch die Lehrkraft selbst eine zentrale Rolle spielen.

## Einstellungen deutscher Lehrkräfte zum Medieneinsatz in den Naturwissenschaften

Dass Medien grundsätzlich das Lehren und Lernen beeinflussen und per se ganz unterschiedliche Funktionen im Lernprozess einnehmen können, ist die Ausgangslage, vor der Unterrichtsplanung und -durchführung stattfinden. Ob und inwiefern entsprechende Potenziale genutzt und ausgeschöpft werden, hängt maßgeblich von den Hauptakteuren der Unterrichtsplanung und -durchführung ab: den Lehrkräften. Es wird davon ausgegangen, dass die Einstellungen einer Lehrkraft ihr Verhalten beeinflussen, also prädiktiv für ihr unterrichtliches Handeln sind (z. B. Dubberke et al. 2008).

Aktuelle Forschungsbefunde zu den Einstellungen deutscher Lehrkräfte zum Medieneinsatz liegen wiederum zunächst unspezifisch im Rahmen des Länderindikators 2017 (Lorenz et al. 2017b) vor. Mit Hilfe von Regressionsanalysen konnten zwei Faktoren als prädiktiv für den Medieneinsatz im Unterricht identifiziert werden. Dies ist zum einen die Einschätzung der Lehrkräfte, dass sie über ausreichend Zeit zur Vorbereitung computergestützten Unterrichts verfügen, und zum anderen die Einstellung, dass der Einsatz von Computern im Unterricht die schulischen Leistungen der Schülerinnen und Schüler verbessert. Probanden, die diesen beiden Punkten zustimmen, verwenden Computer signifikant häufiger in ihrem Unterricht als Lehrkräfte, die diesen beiden Indikatoren ablehnend gegenüberstehen. Ebenfalls fachübergreifend haben Lindau et al. (2013) die Bereitschaft von Lehramtsstudierenden zum Medieneinsatz untersucht. Im Fokus des Forschungsinteresses stand unter anderem der Zusammenhang zwischen den generellen Einstellungen sowie den subjektiv wahrgenommenen Kompetenzen und der Bereitschaft, digitale Medien im Unterricht einzusetzen. Sowohl die Einstellungen als auch die subjektiv wahrgenommenen Kompetenzen konnten in der Untersuchung als signifikante Prädiktoren für die Bereitschaft, digitale Medien im Unterricht einzusetzen, bestätigt werden. Spezifisch für den naturwissenschaftlichen Unterricht betrachten Vogelsang et al. (2019) Lehramtsstudierende für naturwissenschaftliche Fächer und bestätigen die Einstellungen und die Selbstwirksamkeitserwartungen als wichtige Prädiktoren für die motivationalen Orientierungen zum Medieneinsatz. Weiterhin zeigt die oben bereits erwähnte Studie von Pietzner (2009), dass sich in der Gruppe der „Meider“ als Hinderungsgründe sowohl die Ausstattung der Schule (also die Verfügbarkeit) als auch die Unsicherheit im Umgang mit Computern finden.

Insgesamt wurde also festgestellt, dass es für den naturwissenschaftlichen Unterricht Befunde zur Ausstattung mit digitalen Medien gibt und darüber hinaus einzelne Studien zur Mediennutzung, die aber nicht zwischen Fächern differenzieren. Zudem basieren diese teilweise auf Stichproben von naturwissenschaftlichen Lehramtsstudierenden. Dabei ist zu berücksichtigen, dass angehende Lehrkräfte und ausgebildete Lehrkräfte zwar ähnliche Einstellungen zum Einsatz von digitalen Medien haben, ausgebildete Lehrkräfte jedoch äußere Rahmenbedingungen wie die Unterstützung beim Medieneinsatz oder bei der Bereitstellung von Ressourcen als entscheidende Limitationen für den Medieneinsatz benennen (Smarkola 2008), was die Aussagekraft der Studien mit Lehramtsstudierenden einschränkt.

## Ziele und Fragestellungen

Bezüglich der Befunde zur Medienverfügbarkeit und -nutzung fehlt es derzeit an einem präziseren Bild der Situation in den naturwissenschaftlichen Fächern. Bisherige Untersuchungen haben die Medienverfügbarkeit und -nutzung im Allgemeinen oder für größere Fächergruppen erhoben und nicht spezifisch für einzelne Fächer oder Domänen. Erhebungen beziehen sich zudem häufig ohne Berücksichtigung der Klassenebene auf das Nutzungsverhalten der Lehrkräfte allgemein oder sogar nur auf die Nutzungsintention von angehenden Lehrkräften. Für die weitere Entwicklung fachdidaktischer Konzepte unter Einbezug analoger und digitaler Medien ist die Analyse des Ist-Zustandes von hoher Bedeutung, um zum einen den damit einhergehenden Ausstattungsbedarf von Schulen aber auch den Fortbildungsbedarf der Lehrkräfte ermitteln zu können und um zum anderen Einstellungen der Lehrkräfte festzustellen. Mit der oben beschriebenen Heuristik wird eine (fachspezifische) Tiefenstruktur des Medieneinsatzes vorgeschlagen. Es ist zu prüfen, inwieweit dieses theoretische Postulat mit der Unterrichtspraxis in Einklang steht. Ein erster möglicher Zugang ist eine Befragung von Lehrkräften.

Das Ziel wird über drei Zugänge verfolgt: (1) Die Beschreibung der Medienverfügbarkeit und -nutzung im naturwissenschaftlichen Unterricht, (2) die Untersuchung fachdidaktischer Ziele der Mediennutzung durch Lehrkräfte und (3) die Erfassung der Einstellungen und Selbstwirksamkeitserwartungen von Lehrkräften zum Medieneinsatz. Zu den Zielen wurden insgesamt vier Forschungsfragen aufgestellt:

### FF1

Inwiefern sind bestimmte Medien an Schulen in den drei Naturwissenschaften verfügbar und wie zufrieden sind die Lehrkräfte für naturwissenschaftliche Fächer mit dieser Ausstattung?

### FF2

Zu welchen Anteilen werden bestimmte Medien im naturwissenschaftlichen Unterricht genutzt?

### FF3

Welche Einstellungen und welche Selbstwirksamkeitserwartung haben Lehrkräfte für naturwissenschaftliche Fächer hinsichtlich des Medieneinsatzes?

### FF4

Welche Funktionen schreiben Lehrkräfte für naturwissenschaftliche Fächer dem Medieneinsatz bezogen auf exemplarische Medien zu?

Um unter Wahrung einer vernünftigen Befragungslänge die Vergleichbarkeit der Angaben zu gewährleisten, wurde in dieser Studie für alle Fragen eine exemplarische Medienauswahl vorgegeben, die typische Medien umfasst und gleichzeitig einen Fächervergleich zulässt. Gleichwohl ist diese Auswahl keinesfalls vollständig.

Mit Blick auf die Hauptintention der Untersuchung, der Kontrastierung der drei Fächer Biologie, Chemie und Physik und der fachspezifisch unterschiedlichen Mediennutzung wird a posteriori geprüft, ob es differenzielle Effekte gibt.

## Fragebogenerhebung zum Medieneinsatz im naturwissenschaftlichen Unterricht

Zur Beantwortung der Forschungsfragen wurde von April 2017 bis September 2018 eine Fragebogenuntersuchung durchgeführt. Der Fragebogen wurde für Lehrkräfte der Fächer Biologie, Chemie und Physik konzipiert. Eine vergleichbare, teilweise gleiche Fragengestaltung für die fachspezifischen Teile ermöglicht Bezüge zwischen den Befunden in den einzelnen Unterrichtsfächern herzustellen^1^. Die Lehrkräfte wurden sowohl online wie auch offline befragt. Der Kontakt zu den Lehrkräften erfolgte per Email im Rahmen von bestehenden Projekten und Kooperationen sowie auf Tagungen und Weiterbildungen persönlich. Außerdem wurden Schulen mit der Bitte angeschrieben, die Informationen an die Lehrkräfte der mathematisch-naturwissenschaftlichen Fächer weiterzugeben. Da es sich um eine Gelegenheitsstichprobe handelt, ist sie nicht repräsentativ. Lehrkräfte aus den Bundesländern Schleswig-Holstein und Nordrhein-Westfalen sind zum Beispiel deutlich überrepräsentiert (Tab. 1). Jede Lehrkraft konnte den Bogen für eines oder mehrere Fächer ausfüllen. Eine Lehrkraft für Biologie und Chemie konnte den Bogen beispielsweise nur für das Fach Biologie, nur für das Fach Chemie oder für beide Fächer ausfüllen. Im Folgenden konzentriert sich der Beitrag auf diejenigen Lehrkräfte, die den Fragebogen vollständig für ein naturwissenschaftliches Fach ausgefüllt haben, dies trifft insgesamt auf *N* = 189 Personen zu. Die Erhebung der Daten erfolgte vollanonymisiert. In der Stichprobe waren keine Effekte der Schulform, der Berufserfahrung oder des Geschlechts auf die Antworten nachweisbar, wobei dies vor dem Hintergrund der Fallzahlen und verwendeten Skalen auch nicht zu erwarten war. Der Fragebogen ist vollständig als zusätzliches Material online verfügbar.

**Table 1:** 

		Bundesland			Schulform			Berufserfahrung				Geschlecht	
	Häufigkeit	NRW	SH	Sonstiges	Gymnasium	Gesamtschule mit Sek II	Sonstiges	0–5 Jahre	6–10 Jahre	11–20 Jahre	> 20 Jahre	Männlich	Weiblich
Biologie	53	27	12	14	37	13	3	14	12	15	12	19	34
Chemie	54	33	14	7	30	15	9	10	14	15	15	27	27
Physik	82	32	12	38	53	22	7	13	21	20	28	59	23
Gesamt	189	92	38	59	120	50	19	37	47	50	55	105	84

**Figure 1:**
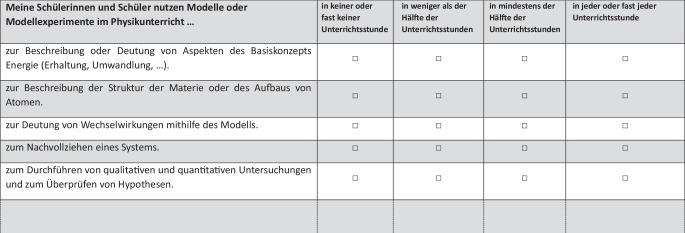

Bezogen auf die drei Fächer bestand der Fragebogen aus mehreren Abschnitten: (1) Zunächst wurden die persönlichen Rahmendaten erfasst. (2) Im Anschluss wurde die Lehrkraft gebeten, sich für ein Fach zu entscheiden. Hier wurde nun je erhoben, in welchem Umfang bestimmte Medien in der Schule zur Verfügung stehen (5-stufig) und wie zufrieden die Lehrkräfte mit dieser Ausstattung sind (4-Punkt Likert-Skala). Die Liste der Medien wurde für die drei Naturwissenschaften im Wesentlichen vergleichbar gehalten. (3) Darüber hinaus sollten die Lehrkräfte für eine durchschnittliche Unterrichtseinheit angeben, wie viele Minuten welches Medium zum Einsatz kommt. Für diese Frage wurde bei der Konstruktion des Bogens eine Gesamtminutenzahl für die Unterrichtseinheit von 360 min vorgegeben. Diese Vorgabe diente der Vergleichbarkeit über alle Lehrkräfte hinweg. Für die exemplarisch vorgegebenen Medien mussten die Lehrkräfte einzeln angeben, wie viele Minuten jedes Medium im Unterricht genutzt wird. Dieses Format berücksichtigt, dass es sowohl Abschnitte geben kann, in denen keines der vorgegebenen Medien genutzt wird, als auch Abschnitte, in denen mehrere Medien gleichzeitig genutzt werden. In der Konsequenz bedeutet das auch, dass die befragten Lehrkräfte mit der Summe ihrer Zeitangaben je Medium diese Gesamtzeit unter- bzw. überschreiten oder genau ausfüllen konnten. (4) Abschließend sollten die Lehrkräfte für drei exemplarische Medien angeben, wie häufig sie das jeweilige Medium im Unterricht nutzen, um eine bestimmte Funktion zu erfüllen (Abb. 1). Als exemplarische Medien wurden gegenständliche Modelle, Computerhardware (PC, Laptop, Beamer, etc.) und virtuelle Lernumgebungen gewählt. Als Funktionen wurden Kompetenzen in Anlehnung an die Bildungsstandards formuliert, die sich auch als Tätigkeiten verstehen lassen. Die Befragung sollte damit einerseits anschlussfähig an den Schulalltag der Lehrkräfte sein, andererseits die Beantwortung der Frage ermöglichen, ob bestimmte Medien spezifische fachdidaktische Potenziale haben, auf die über die Funktionen geschlussfolgert werden kann. Diese Auswahl umfasst bewusst analoge und digitale sowie mehr oder weniger geläufige Medien.^2^ (5) Ferner wurden die Einstellungen der Lehrkräfte gegenüber dem Unterrichten mit digitalen Medien erfasst (Lindau et al. 2013). Die Lehrkräfte sollten ihre Zustimmung zu sechs Aussagen auf einer fünf-stufigen Likert-Skala beurteilen (von 1: stimme überhaupt nicht zu bis 5: stimme voll zu). Die Selbstwirksamkeitserwartung in Bezug auf die Planung und Durchführung von Unterricht mit digitalen Medien wurde mithilfe von sechs Items abgefragt (Adaption der Skala „Experimentieren“ für digitale Medien nach Meinhardt et al. 2016; z.  B. „Ich kann den Einsatz eines digitalen Mediums im Fachunterricht didaktisch begründen, auch wenn ich dieses digitale Medium noch nicht selbst eingesetzt habe.“). Die Antworten wurden mit einem vierstufigen Antwortformat erfasst (von 1: stimmt genau bis 4: stimmt nicht).

## Ergebnisse der Fragebogenerhebung

Bezogen auf die Verfügbarkeit von Medien und die Zufriedenheit mit der Ausstattung muss einschränkend erwähnt werden, dass in dieser Befragung exemplarisch nach drei ausgewählten Medien gefragt wurde: gegenständliche Modelle, Computerhardware und virtuelle Lernumgebungen. Zur Analyse der Likert-Skalen wurde, De Winter und Dodou (2010) folgend, auf parametrische Testverfahren zurückgegriffen. Sie konnten zeigen, dass sich non-parametrische und parametrische Verfahren in den Resultaten nur in sehr speziellen Fällen unterscheiden, die hier nicht vorliegen. Zur Sicherheit wurden gefundene Unterschiede mit non-parametrischen Verfahren geprüft. Folglich wurden paarweise t‑Tests zur Bestimmung von Fachunterschieden berechnet. Es zeigt sich, dass bei der Verfügbarkeit Fachspezifika nur für Hardware (zwischen Biologie und Chemie) sowie für virtuelle Lernumgebungen (zwischen Physik und beiden anderen Fächern) auftreten (Tab. 2 und 3), wobei unklar ist, ob dies von der Fachkultur oder einer fachspezifischen Ausstattung abhängt. Es wird zudem deutlich, dass gegenständliche Modelle als Sammlungsgegenstand der Lehrkraft zur Verfügung stehen; darüber hinaus ist die Hardware insbesondere nicht immer für alle Lernenden verfügbar (was sich auch auf die Verfügbarkeit der virtuellen Lernumgebungen niederschlagen dürfte). Für die Zufriedenheit lässt sich zusammenfassen, dass es kaum fachspezifische Befunde gibt und die Lehrkräfte mit der analogen Ausstattung eher zufriedener sind, als mit der digitalen.

**Table 2:** 

Verfügbarkeit exemplarischer Medien in den drei naturwissenschaftlichen Fächern (% je Fach und Kategorie)															
	Modelle & Modellexperimente					*PC & Laptop*^*a*^					*Virtuelle Lernumgebung*^*b,c*^				
	Für alle Lernenden immer verfügbar	Bei Bedarf als mobiler Klassensatz verfügbar	Bei Bedarf im Fachraum verfügbar	Bei Bedarf einzelne verfügbar	Nicht verfügbar	Für alle Lernenden immer verfügbar	Bei Bedarf als mobiler Klassensatz verfügbar	Bei Bedarf im Fachraum verfügbar	Bei Bedarf einzelne verfügbar	Nicht verfügbar	Für alle Lernenden immer verfügbar	Bei Bedarf als mobiler Klassensatz verfügbar	Bei Bedarf im Fachraum verfügbar	Bei Bedarf einzelne verfügbar	Nicht verfügbar
Biologie	7,5	3,8	20,8	67,9	0	3,8	23,1	34,6	28,8	9,6	1,9	9,4	30,2	3,8	47,2
Chemie	11,3	3,8	30,2	50,9	3,8	13,2	26,4	39,6	15,1	5,7	3,9	13,7	29,4	17,6	35,3
Physik	9,9	1,2	33,3	51,9	3,7	26,5	19,1	2,9	27,9	23,5	8,9	12,7	55,7	7,6	15,2

*Hinweis*: In jeder Zelle ist angegeben, wie viele Prozent der Lehrkräfte die jeweilige Option auf der 5‑stufigen Skala ausgewählt haben: Zum Beispiel gaben 7,5 % der Biologielehrkräfte an, dass Modelle für alle Lernenden immer verfügbar sind. Für etwaige Fachspezifika wurde für die 5‑stufigen Skalen immer zwischen zwei Fächern paarweise verglichen, ob die Lehrkräfte sich in ihrem Antwortverhalten unterscheiden. Es sind die Spaltenüberschriften derjenigen Spalten kursiv gedruckt, in denen es zu Fachunterschieden kommt:

^a^ sign. zwischen Biologie & Chemie

^b^ sign. zwischen Biologie & Physik

^c^ sign. zwischen Chemie & Physik

**Table 3:** 

Zufriedenheit exemplarischer Medien in den drei naturwissenschaftlichen Fächern (% je Fach und Kategorie)												
	Modelle & Modellexperimente				PC & Laptop				*Virtuelle Lernumgebung*^*a*^			
	Sehr zufrieden	Eher zufrieden	Eher unzufrieden	Sehr unzufrieden	Sehr zufrieden	Eher zufrieden	Eher unzufrieden	Sehr unzufrieden	Sehr zufrieden	Eher zufrieden	Eher unzufrieden	Sehr unzufrieden
Biologie	13,2	54,7	28,3	3,8	6,1	36,7	46,9	10,2	4	20	64	12
Chemie	10	44	44	2	13,5	38,5	34,6	13,5	13,3	23,3	50	13,3
Physik	7,9	53,9	30,3	7,9	12,7	30,2	34,9	22,2	17,6	41,2	35,3	5,9

*Hinweis*: In jeder Zelle ist angegeben, wie viele Prozent der Lehrkräfte die jeweilige Option auf der Likert Skala ausgewählt haben: Zum Beispiel gaben 13,2 % der Biologielehrkräfte an, mit der Ausstattung an Modellen sehr zufrieden zu sein. Für etwaige Fachspezifika wurde für die Likert Skalen immer zwischen zwei Fächern paarweise verglichen, ob die Lehrkräfte sich in ihrem Antwortverhalten unterscheiden. Es sind die Spaltenüberschriften derjenigen Spalten kursiv gedruckt, in denen es zu Fachunterschieden kommt

^s^ sign. zwischen Biologie & Physik

Zur Beantwortung der zweiten Forschungsfrage wurden die Lehrkräfte gebeten anzugeben, welches Medium von ihnen wie lange genutzt wird. Forschungsfrage zwei fokussiert daher auf den Vergleich der relativen Nutzung der Medien untereinander, wobei zu bedenken ist, dass manche Kombinationen (virtuelle Lernumgebung und Hardware) nicht trennbar sind.

Um dieses Ziel zu verfolgen, wurde zunächst die Mediennutzungszeit je Person normiert (normierte Zeit Medium x, Lehrkraft y = Zeit für Medium x bei Lehrkraft y/Summe der Zeit aller Medien bei Lehrkraft y), um auf der Basis der normierten Zeit je Lehrkraft anschließend über die Lehrkräfte hinweg eine mittlere Nutzung eines bestimmten Mediums angeben zu können (mittlere Zeit Medium x = Mittelwert aller mittleren Zeiten Medium x über alle Lehrkräfte). Darauf aufbauend kann der Anteil dieses Mediums im Vergleich zu allen Medien verglichen werden. Diese Anteile beziehen sich also je Lehrkraft ausdrücklich nur auf die relative Zeit, in der diese Lehrkraft die hier abgefragten Medien nutzt.

Insgesamt muss festgehalten werden, dass es sehr hohe Unterschiede zwischen einzelnen Lehrkräften gibt (s. Standardabweichungen), sowohl hinsichtlich der gesamten Mediennutzungszeit als auch der Verhältnisse der einzelnen Medien zueinander. Diese Unterschiede zwischen den Personen eines Fachs übertreffen auch die Unterschiede zwischen den Fächern. Dies ist insofern wichtig, als dass zusätzlich ein Vergleich der Fächer fachspezifische Mediennutzungsmuster aufdecken soll (Tab. 4). Erwartungskonform zeigt sich, dass gegenständliche Modelle im Biologieunterricht mehr Zeit in Anspruch nehmen. Der zeitliche Anteil von Experimenten fällt zwischen allen Fächern unterschiedlich aus, anteilig ist die Rolle im Chemieunterricht am größten (~40 %), es folgen der Physik- (~32 %) und der Biologieunterricht (~21 %). Hier können fachdidaktisch begründbare Muster gesehen werden, wohingegen die Unterschiede bei Projektor, Tafel und Schulbuch zunächst theoretisch schwer greifbar sind.

**Table 4:** 

Mediennutzung in den drei Fächern (Prozent der Gesamtmediennutzungszeit gemittelt über die Personen)													
		*Ein gegenständliches Modell*^*a,b*^	Eine virtuelle Lernumgebung	*Ein reales Experiment*^*a,b,c*^	Ein Smartphone	Einen Tablet-PC	Ein Notebook	Einen Computer/Desktop PC	*Einen Overhead-Projektor*^*a,b*^	*Eine interaktive Tafel*^*a,b*^	*Ein Schulbuch*^*a,b*^	Cassy, Cobra o. ä.	Einen graphischen Taschenrechner (mit Sensoren)
Biologie	Mittelwert	16,28	3,46	21,05	1,67	2,25	5,99	7,16	9,76	4,11	24,51	n.a.	n.a.
	Stand.abw	11,30	6,96	13,00	3,80	8,02	13,20	11,14	11,00	10,80	17,73	n.a.	n.a.
Chemie	Mittelwert	7,20	3,66	40,48	3,31	2,25	4,55	4,99	3,79	10,58	17,33	n.a.	n.a.
	Stand.abw	7,64	7,18	19,20	5,71	7,24	10,06	8,50	7,10	16,01	17,41	n.a.	n.a.
Physik	Mittelwert	9,85	4,76	32,26	2,37	2,44	2,77	8,17	2,69	13,73	13,75	1,36	2,69
	Stand.abw	11,35	8,93	20,32	5,07	9,97	6,58	12,31	5,32	23,44	13,49	5,31	5,32

*Hinweis*: Bestimmt wurde ausgehend von der gesamten Mediennutzungszeit je Lehrkraft der prozentuale Anteil bestimmter Medien an dieser Nutzungszeit. Damit bleiben Zeiten unberücksichtigt, in denen keines der hier abgefragten Medien genutzt wird. Ferner werden sich manche Zeiten doppeln, so kann ein reales Experiment zum Beispiel mit einem Smartphone durchgeführt oder ein Schulbuch auf einem Tablet gelesen werden. Aufgrund der Berechnungsgrundlage ist dies nicht ersichtlich. Es lässt sich demnach nur ein relativer Vergleich ablesen: Es nehmen zum Beispiel in einer typischen Biologie Unterrichtsreihe durchschnittlich reale Experimente mehr Zeit in Anspruch als die Arbeit mit gegenständlichen Modellen. Oder für den Umgang mit gegenständlichen Modellen wird im Biologieunterricht im Durchschnitt mehr Zeit genutzt als im Chemieunterricht. Auf letzteres beziehen sich auch die Signifikanztests: Überprüft wurden paarweise Vergleiche für die normierten Anteile je Medium immer zwischen zwei Fächern. Kursiv gedruckt sind Spaltenüberschriften von denjenigen Spalten mit signifikanten Unterschieden

^a^ sign. zwischen Biologie & Chemie

^b^ sign. zwischen Biologie & Physik

^c^ sign. zwischen Chemie & Physik

*n.a.* die Nutzungszeiten wurden im Fragebogen des jeweiligen Faches nicht erhoben

Im Fragebogen gab es zusätzlich Fragen zur Einstellung der Lehrkräfte bezogen auf die Nutzung digitaler Medien im Unterricht (allgemein) und Fragen zur Selbstwirksamkeitserwartung bezogen auf Planung und Durchführung von Unterricht mit digitalen Medien (allgemein). Die Skalenreliabilitäten der Likert-Skalen zur Selbsteinschätzung waren angemessen (Selbstwirksamkeitserwartung, 6 Items, Cronbach’s *α* = 0,772, *N* = 178; Einstellung gegenüber digitalen Medien, 6 Items, Cronbach’s *α* = 0,796, *N* = 178). Der Median für die Skala der Selbstwirksamkeitserwartung liegt bei 3 (auf einer vierstufigen Likert-Skala), der Median für die Einstellungsskala bei 4 (auf einer fünfstufigen Likert-Skala). Beide Verteilungen zeigen leichte Deckeneffekte. Wie bereits bei den Analysen zur Zufriedenheit und Verfügbarkeit werden auch hier nach De Winter und Dodou (2010) parametrische Testverfahren angewendet. Zur Sicherheit wurden gefundene Unterschiede über non-parametrische Verfahren überprüft. Folglich wurden paarweise t‑Tests zur Bestimmung von Fachunterschieden berechnet. Für die Untersuchung des Zusammenhangs zwischen den beiden Skalen wurden nicht-parametrische Korrelationen genutzt.

Es zeigten sich keine statistisch bedeutsamen Unterschiede für die Selbstwirksamkeitserwartung und die Einstellung zur Nutzung digitaler Medien im Unterricht zwischen den Fächern, wobei über alle Lehrkräfte und Fächer hinweg die Skalen in mittlerer Höhe korrelieren (Spearman’s *ρ* = 0,426; *p* < 0,001). Zusätzlich war von Interesse, ob sich ein Zusammenhang zwischen der Zufriedenheit mit der Ausstattung bezogen auf Hard- oder Software für den naturwissenschaftlichen Unterricht und der Selbstwirksamkeitserwartung bzw. der Einstellung gegenüber digitalen Medien zeigt. Hier findet sich jedoch kein statistisch bedeutsamer Zusammenhang.

Um die vierte Forschungsfrage zu beantworten, wurde für die drei ausgewählten Medien wieder mit Blick auf den Unterricht in einer Klasse gefragt, mit welchen Zielen sie genutzt werden. Hierfür kam eine vierstufige Skala zum Einsatz, mit den Kategorien: (1) in keiner oder fast keiner Unterrichtsstunde, (2) in weniger als der Hälfte der Unterrichtsstunden, (3) in mindestens der Hälfte der Unterrichtsstunden, (4) in jeder oder fast jeder Unterrichtsstunde. Es muss eingeschränkt werden, dass in der Praxis in einer Stunde ein Medium auch verschiedene Funktionen erfüllen kann, was hier nicht erkennbar ist.

Bei der Betrachtung der Rohdaten zeigt sich für manche Funktionen innerhalb einer Fachgruppe eine deutliche Schiefe in der Verteilung. Aus diesem Grund wurden nun ausschließlich Mediane berichtet und non-parametrische Verfahren gerechnet (vgl. De Winter und Dodou 2010). Es ist ferner wichtig zu betonen, dass aufgrund der sehr niedrigen Anteile digitaler Medien generell (vgl. Tab. 4) ein Blick nur auf die gegenständlichen Modelle aussagekräftig ist, für die sich auch – durchaus erwartbare – Fachspezifika zeigen (vgl. Tab. 5 und 6). Eine fachdidaktische Interpretation der Muster ist zum Beispiel, dass gegenständliche, nicht prozesshafte Modelle in der Physik kaum genutzt werden (in Biologie ein Modell der Haut, in Chemie ein Modell eines Moleküls), daher werden in der Physik Modelle auch häufiger herangezogen, um „reale“ Experimente durchzuführen und Daten zu erheben. Eine reine Beschreibung kommt seltener vor.

**Table 5:** 

Ziel der Nutzung von Modellen im naturwissenschaftlichen Unterricht																			
		*Untersuchungen durchführen*^*b,c*^	*Daten erheben*^*b,c*^	*Regeln herausfinden*^*b,c*^	*Strukturen analysieren*^*a,b,c*^	Fragen beantworten	Veranschaulichen	*Modell reflektieren*^*c*^	*Modellgrenzen diskutieren*^*a*^	Aufbau & Funktion von Molekülen, Zellen, Organismen	Deutung von Strukturbeziehungen	Vergleich Anatomie und Morphologie	Beschreibung Aufbau von Stoffen und Atomen	Deutung von Stoffeigenschaften auf Teilchenebene	Deutung von Stoff- und Energieumwandlungen	Beschreibung oder Deutung Basiskonzept Energie	Beschreibung Struktur der Materie	Deutung von Wechselwirkungen	Nachvollziehen eines Systems
Biologie	Median	2	2	2	2	2	3	2	2	3	2	2	n.a.	n.a.	n.a.	n.a.	n.a.	n.a.	n.a.
Chemie	Median	2	1	1	1	2	3	2	2	n.a.	n.a.	n.a.	2	2	2	n.a.	n.a.	n.a.	n.a.
Physik	Mediean	2	2	2,5	2	3	3	2	2	n.a.	n.a.	n.a.	n.a.	n.a.	n.a.	2	2	2	2

*Hinweis*: In den Tabelleneinträgen findet sich je Fach und Funktion der Median über die vier Stufen der 4‑stufigen Skala: (1) in keiner oder fast keiner Unterrichtsstunde, (2) in weniger als der Hälfte der Unterrichtsstunden, (3) in mindestens der Hälfte der Unterrichtsstunden, (4) in jeder oder fast jeder Unterrichtsstunde. Kursiv gedruckt sind Funktionen, bei denen es zu Unterschieden in der Einschätzung zwischen jeweils zwei Fächern kommt

^a^sign. zwischen Biologie & Chemie

^b^sign. zwischen Biologie & Physik

^c^sign. zwischen Chemie & Physik

*n.a.* die Nutzungen wurden im Fragebogen des jeweiligen Faches nicht erhoben

**Table 6:** 

Ziel der Nutzung von PC/Laptop im naturwissenschaftlichen Unterricht												
		Erstellen von Präsentationen	Recherche	Kommunikation	Leistungsüberprüfung	Übung	Simulieren & Modellieren	*Messen*^*a,b,c*^	Video & Bildbearbeitung	Zeichnung von Strukturen & Versuchen	Berechnungen	Tabellen & Diagramme
Biologie	Mittelwert	1,94	2,12	1,32	1,38	1,54	1,58	1,20	1,36	1,18	n.a.	n.a.
	Stand.abw	0,85	0,85	0,79	0,67	0,68	0,73	0,45	0,69	0,56	n.a.	n.a.
Chemie	Mittelwert	1,83	2,15	1,43	1,29	1,62	1,60	1,31	1,38	1,29	n.a.	n.a.
	Stand.abw	0,76	0,78	0,78	0,61	0,77	0,63	0,54	0,63	0,54	n.a.	n.a.
Physik	Mittelwert	1,70	2,13	1,66	1,29	n.a.	1,67	2,07	n.a.	n.a.	2,39	1,86
	Stand.abw	0,74	0,96	1,08	0,65	n.a.	0,73	0,86	n.a.	n.a.	1,11	0,91

*Hinweis: *Es handelt sich um eine vierstufige Skala mit den Kategorien: (1) in keiner oder fast keiner Unterrichtsstunde, (2) in weniger als der Hälfte der Unterrichtsstunden, (3) in mindestens der Hälfte der Unterrichtsstunden, (4) in jeder oder fast jeder Unterrichtsstunde

*n.a.* die Nutzungen wurden im Fragebogen des jeweiligen Faches nicht erhoben

^a^ sign. zwischen Biologie & Chemie

^b^ sign. zwischen Biologie & Physik

^c^ sign. zwischen Chemie & Physik

## Limitationen der Untersuchung

Eine entscheidende Limitation der Untersuchung ergibt sich aus der Rekrutierung der Teilnehmenden. Diese wurden per Email oder auf Tagungen angesprochen und konnten freiwillig an der Befragung teilnehmen. Damit handelt es sich um eine Gelegenheitsstichprobe in der Variante Selbstselektionsstichprobe. Weniger motivierte Personen haben vermutlich aufgrund der fehlenden persönlichen Einladung nicht an der Befragung teilgenommen. Damit wird der Medieneinsatz gemessen an der Nutzungshäufigkeit in der vorliegenden Untersuchung vermutlich überschätzt. Nahezu alle Teilnehmenden stammen aus den Bundesländern Nordrhein-Westfalen und Schleswig-Holstein. Da insbesondere im Kontext des Länderindikators 2017 mitunter große Unterschiede in der Nutzung von Medien für konkrete Funktionen zwischen den Bundesländern festgestellt wurden, kann auf Basis dieser Stichprobe nicht mit Blick auf das gesamte Bundesgebiet generalisiert werden. Es kann nur indirekt auf die Inferenzpopulation geschlossen werden. Ferner muss bedacht werden, dass nahezu alle Befragten an Schulformen mit Oberstufe unterrichten und dass damit Schulen, die zum Mittleren Schulabschluss führen, unterrepräsentiert sind.

Eine weitere Limitation betrifft die Auswahl dreier Medien, zu denen die Lehrkräfte mit Blick auf deren Funktion befragt wurden. Bei der Wahl der Medien wurden bewusst analoge und digitale sowie eher typische und weniger typische gewählt. Darüber hinaus ist die Auswahl der Medien auch durch den fächervergleichenden Charakter des Gesamtprojekts geprägt: Neben den Naturwissenschaften fanden parallele Befragungen auch in der Mathematik und an außerschulischen Lernorten statt. Da ein Projektziel auch der Vergleich zu diesen Erhebungen war, wurden solche Medien gewählt, die mögliche Fachspezifika andeuten. Nicht zuletzt war die Auswahl der Medien auch durch die Befragungszeit begrenzt. Dadurch wurde jedoch gleichzeitig eine Engführung vorgenommen, durch die die Lehrkräfte nur spezifisch zu diesen Medien befragt wurden. Es kann nicht ausgeschlossen werden, dass die Lehrkräfte intensivere Erfahrungen mit der Nutzung anderer Medien gemacht haben und somit spezifischer konkrete Funktionen hätten benennen können. Die gezielte Fokussierung auf wenige beispielhafte Medien hat allerdings den Vorteil, dass dadurch ein Vergleich zwischen den drei naturwissenschaftlichen Fächern Biologie, Chemie und Physik möglich wird. Nichtsdestoweniger: Bei den Resultaten zur Nutzungszeit bestimmter Medien in einer Unterrichtsreihe lässt sich nicht ausschließen, dass es weitere Medien gegeben hätte, die Lehrkräfte hier aufführen würden. Auch bei den Funktionen der drei exemplarisch gewählten Medien zeigt sich durch die geringe relative Häufigkeit digitaler Medien im naturwissenschaftlichen Unterricht, dass zwei Fragen durch die Medienwahl (Funktion von Hardware, Funktion von virtuellen Lernumgebungen) nicht belastbar waren. Es sei an dieser Stelle nur erwähnt, dass sich dies für außerschulische Lernorte und für Mathematikunterricht tatsächlich anders darstellt (Härtig et al. 2020; Kampschulte et al. 2019). Es bleibt festzuhalten: Aufgrund dieser Auswahl und der damit verbundenen Beschränkung kann die Befragung keinen validen Aufschluss über einzelne Medien geben, sondern höchstens hypothesengenerierend genutzt werden. Dem Ziel eines Fächervergleichs jedoch ist dies nicht abträglich, weil alle diskutierten Einschränkungen alle drei Fächer gleichermaßen betreffen.

Spezifisch für die Frage nach den Zeitanteilen bestimmter Medien an der Unterrichtszeit muss kritisch diskutiert werden, inwieweit die Frage selbst die Ergebnisse beeinflusst hat. In den Ergebnissen deutet sich an, dass die Mehrheit der Lehrkräfte bei ihren Angaben versucht hat, in der Summe exakt auf die vorgegebene Gesamtzeit zu kommen, andere unter- oder überschreiten diese Zeit (mitunter erheblich). Im Nachhinein lässt sich nicht aufklären, ob dies wirklich an der Art der Fragestellung liegt oder eine andere Formulierung der Frage zu vergleichbaren Resultaten geführt hätte. Insofern sind die absoluten Angaben wenig aussagekräftig und die Analysen müssen sich ausschließlich auf den relativen Anteil eines Mediums an der gesamten Mediennutzungszeit beschränken.

Abschließend ist bei den Limitationen der Zeitpunkt der Befragung zu nennen. Dieser liegt zeitlich vor der Umsetzung der vom Bund angestoßenen Initiative zur Ausstattung von Schulen mit digitaler Technik. Damit haben die derzeit in vielen Bereichen stattfindende Stärkung der Bildung in der digitalen Welt und der damit verbundene Ausbau der Ausstattung von Schulen mit Medien noch keinen Niederschlag in den hier berichteten Ergebnissen gefunden. Die Aktualität der Ergebnisse ist folglich zu hinterfragen, gerade vor dem Hintergrund der im Vergleich zu anderen pädagogischen Innovationen schnellen Entwicklung im Bereich Digitalisierung, die durch die spezielle Situation der Schulen während der COVID-19-Pandemie noch einmal weiteres Momentum dazugewonnen hat.

## Diskussion der Befunde

In der Summe bestätigt die Untersuchung das Bild aus den eingangs erwähnten fachübergreifenden Studien, dass digitale Medien bis heute trotz vieler Diskussionen und konkreter Vorschläge seitens der Fachdidaktik kaum Einzug in den naturwissenschaftlichen Unterricht gehalten haben. Darüber hinaus deuten sich fachspezifische Nutzungshäufigkeiten und Intentionen an, die durchaus im Einklang mit den Ergebnissen von Pietzner (2009) zu Computern stehen und die in der Zukunft näher in den Blick genommen werden sollten (vgl. Tab. 4). Im Folgenden möchten wir diese Befunde detailliert diskutieren und schließlich Implikationen für Forschung und Praxis ableiten. Dabei orientieren wir uns hier aus Gründen der Argumentationslogik nicht an der Reihenfolge der Forschungsfragen.

Ein Ziel der Erhebung war ein erster fachspezifischer Blick auf die Bedeutung bestimmter Medien für den Fachunterricht aus Sicht der Lehrkräfte. Dazu wurde die Nutzungsdauer der verfügbaren Medien analysiert. Zu diesem Zweck wurde für ausgewählte Medien deren Nutzungszeit in einer Unterrichtseinheit erhoben. Dies erlaubt eine relative Rangordnung der Medien untereinander je Fach. Bei der Konzeption des Fragebogens war intendiert, analoge und digitale Medien zu berücksichtigen, um einerseits Fachtraditionen abbilden zu können und andererseits anschlussfähig an aktuelle Untersuchungen zu sein. Ferner sollte die Liste in gewisser Weise praxistauglich sein, also Lehrkräften die Medien in einer Form anbieten, die sich mit typischen Bezeichnungen deckt. Mit Blick auf das Ziel des ausschließlich relativen Vergleichs wurde in Kauf genommen, dass die Liste zu Überlappungen führt. Allerdings sind die Fragen für alle drei Fächer in weiten Teilen gleich gehalten, so dass ein Fächervergleich und damit die Suche nach Fachspezifika möglich wird.

Insgesamt fällt zunächst auf, dass die Nutzungszeiten der digitalen Medien im Vergleich zu den Zeiten der analogen Medien deutlich geringer ausfallen, obwohl digitalen Medien aufgrund ihrer vielfältigen Potenziale eine hohe Nutzungsdauer zugesprochen werden könnte (Blömeke 2003; Herzig und Grafe 2006; Kerres 2000; McKnight et al. 2016; Schanze und Girwidz 2018). Wird die Vielzahl an fachdidaktischen Projekten betrachtet, die seit geraumer Zeit auch fachspezifische Vorschläge für die Unterrichtspraxis liefern, entsteht für alle drei Fächer grundsätzlich der Eindruck einer Theorie-Praxis-Lücke. Diese kann hier nicht weiter aufgeklärt werden, da der Fokus der Untersuchung ein anderer ist. Aber dies hatte zur Folge, dass Teile der Erhebung nicht erfolgreich waren: Die Suche nach fachspezifischen Funktionen von virtuellen Lernumgebungen und generell digitalen Medien (über die Hardware als Zugang) konnten nicht belastbar ausgewertet werden, weil die Nutzungszahlen schlicht zu gering sind. Es kann nur ein erstes Indiz gesammelt werden, über den Anteil digitaler Medien im Rahmen der Gesamtnutzungszeit, wobei diese in allen drei Fächern klar hinter traditionellen Medien rangieren. Es zeigt sich, dass bestimmte Medien offensichtlich im Unterricht mancher naturwissenschaftlicher Fächer häufiger genutzt werden, als in anderen. Fachdidaktisch gut begründbar ist der Befund, dass gegenständliche Modelle insbesondere im Fach Biologie genutzt werden, während Experimente vor allem in der Chemie und in einem etwas geringeren Umfang in der Physik genutzt werden. Die Bedeutung vornehmlich quantitativer Experimente für den Physikunterricht ist plausibel, insbesondere bei einem Blick in Lehrpläne. Eine mögliche Interpretation wäre, dass im Biologieunterricht, verglichen zum Physikunterricht zum Beispiel, ein stärkerer Fokus auf der Verdeutlichung von Strukturen und Funktionen liegt, was sich mit gegenständlichen Modellen gut umsetzen lässt. Die Chemie ist zwischen den beiden anderen Fächern verortet, weil sie – je nach Teilgebiet – beide Ziele verfolgt. Es zeigen sich zwar weitere Unterschiede. Diese erscheinen jedoch schwer erklärbar. In Summe finden sich damit aber Indizien für eine Praxisrelevanz der Heuristik im Sinne fachspezifischer Mediennutzung.

Um dies näher zu untersuchen, lohnt der Blick auf die konkrete Funktion der Medien. Seitens der Mediendidaktik und der Naturwissenschaftsdidaktiken wurden eine Reihe von möglichen Funktionen des Medieneinsatzes für das Lehren und Lernen theoretisch abgeleitet. Untersucht wurde für ganz konkrete Beispiele, z. B. Modelle, welche Funktionen Lehrkräfte diesen zuschreiben (vgl. Tab. 5). Aufgrund der für uns überraschenden, sehr niedrigen Anteile digitaler Medien bleibt nur festzustellen, dass die zwei Fragen zu digitalen Zugängen zu nicht belastbaren Daten führen. In den Daten für Modelle deuten sich aber die erwarteten Unterschiede hinsichtlich der gewählten Funktionen an, wobei die befragten Lehrkräfte angeben, dass sie alle vorgegebenen Funktionen von Medien in ihrem Unterricht einsetzen. Für das Fach Biologie kann zum Beispiel spezifisch festgehalten werden, dass Modelle etwas häufiger zur Diskussion von Modellgrenzen eingesetzt werden. Auffallend ist ferner, dass Modelle insbesondere im Fach Physik bei „eine Untersuchung durchführen“, „Daten erheben“ und „Regeln herausfinden“ genutzt werden, während diese drei Funktionen in den beiden anderen Fächern eine nachgeordnete Rolle einnehmen. Damit kann erstmals ergänzend zu bestehenden Befunden (vgl. Eickelmann et al. 2017; Analyseeinheit Lehrkräfte) ein noch differenzierterer Einblick in Funktionen von Medien im naturwissenschaftlichen Unterricht gegeben werden. Dies beschränkt sich zwar hier auf gegenständliche Modelle, liefert nichtsdestoweniger aber einen Beleg für eine fachspezifische Tiefenstruktur des Medieneinsatzes, der sich in den Nutzungszeiten niederschlägt.

Wenn sich die Funktion bestimmter Medien fachspezifisch darstellt, ist der Zusammenhang zur Nutzungszeit jedoch nur eine mögliche Erklärung. Viel grundlegender muss zunächst dieses Medium verfügbar sein, sonst wird es auch nicht zum Einsatz kommen. Aus diesem Grund wurden Daten zur FF1 erhoben, die die Verfügbarkeit von Medien in Schulen sowie die Zufriedenheit der Lehrkräfte mit der Ausstattung thematisiert. Dies erfolgte fragebogenlogisch für die drei Medien, für die die Funktionen detailliert erfragt wurden. Für die gegenständlichen Modelle, für die auch die anderen Fragen belastbare Befunde liefern, deutet sich an, dass diese grundsätzlich in Schulen in allen drei Fächern zur Verfügung stehen und dass die befragten Lehrkräfte mit der Ausstattung grundsätzlich zufrieden sind. Hieraus lässt sich mit Blick auf die Heuristik schlussfolgern, dass sich ein Unterschied der Oberflächenstruktur des Fachunterrichts bei der Nutzung von Modellen nicht allein durch die Verfügbarkeit von Medien aufklären lässt. Darüber hinaus können die Ergebnisse als Indiz dafür gesehen werden, dass auch die Lehrkräfte zumindest implizit eine Tiefenstruktur des Medieneinsatzes in bestimmten Fällen im Sinne der Heuristik anstreben.

Wenn auch eine Verbindung zu den Funktionen digitaler Medien nicht möglich ist, bleibt doch die Frage, ob die geringen Nutzungszeiten sich fachlich oder durch die grundsätzliche Ausstattung der Schulen begründen. Hier deutet sich in der Tendenz eine Unzufriedenheit der Lehrkräfte mit der Ausstattung mit PC und Notebooks sowie mit virtuellen Lernumgebungen an. Zwar sind PC und Notebooks grundsätzlich vorhanden, doch wünschen sich die Lehrkräfte hier offensichtlich PC und Notebooks für möglichst viele Lernende. Die Existenz von Computerräumen und Tabletkoffern scheint in der Praxis für den naturwissenschaftlichen Fachunterricht nicht hinreichend zu sein. Und da die Hardware schon nicht im Fachraum für alle Lernenden zur Verfügung steht, ist nachgeordnet auch verständlich, dass virtuelle Lernumgebungen deutlich weniger verbreitet sind und Lehrkräfte sich hier eine grundsätzliche Ausstattung von Schulen mit diesem Medium wünschen. Auch dieser Befund passt zu bisherigen Untersuchungen, die aufgrund von Befragungen eine Verbesserung der Ausstattung von Schulen mit Medien anmahnen. Es bleibt anzumerken, dass damit an dieser Stelle nur eine recht allgemeine Aussage im Sinne der Oberflächenstruktur und nicht der Tiefenstruktur der Heuristik getroffen werden kann.

Bezüglich der Ausstattung der Schulen mit Medien und der Zufriedenheit der Lehrkräfte mit dieser Ausstattung zeigen sich Unterschiede zwischen den Lehrkräften der drei Fächer. Im Fach Physik scheinen gegenständliche Modelle als Medium eine weniger starke Bedeutung einzunehmen. Dafür scheinen per se zahlenmäßig mehr Notebooks zur Verfügung zu stehen. Trotzdem sind die befragten Lehrkräfte mit der Ausstattung überwiegend unzufrieden. Das dritte Medium, virtuelle Lernumgebungen, ist im Vergleich zu den beiden anderen Medien eher selten verfügbar. Am ehesten können Physiklehrkräfte auf diese zugreifen. Biologie- und Chemielehrkräfte sind überwiegend unzufrieden mit der Ausstattung und wünschen sich hier eine Verbesserung. Die berichteten Zahlen ergänzen bisherige Befunde zur Medienausstattung und geben erstmals einen Einblick in die Situation einzelner Fächer. Offensichtlich unterscheiden sich insbesondere die Ausstattungen zwischen den Fächern Biologie und Physik.

## Desiderata

Die vorliegende Befragung gibt einen Einblick in die Verknüpfung von Medienverfügbarkeit und -einsatz sowie die Kopplung des Einsatzes an bestimmte Funktionen aus Sicht der Lehrkräfte. Eine solche Kopplung von Medien an bestimmte Funktionen stellt eine fachdidaktische Tiefenstruktur des Medieneinsatzes dar, wie sie die eingangs dargestellte Heuristik postuliert. Die Idee der Heuristik ist an dieser Stelle, dass ganz bestimmte Medien mehr oder weniger zuträglich zum Verfolgen und Erreichen bestimmter Lernziele sind. Dies wird in der Heuristik als das fachdidaktische Potenzial des Mediums bezeichnet. Aus Sicht der Lehrkraft ist das Potenzial mit der hier erfragten Funktion verknüpft, die deswegen als kompetenzorientiertes Lernziel formuliert wurde. Die vorliegende Untersuchung konnte am Beispiel der gegenständlichen Modelle ein erstes Indiz aufdecken, dass auch Lehrkräfte zumindest implizit bestimmten Medien fachdidaktisch spezifische Funktionen zuschreiben. Diese noch sehr vage Verknüpfung gilt es präziser zu untersuchen. Mit Blick auf die Unterrichtspraxis fehlen insbesondere Untersuchungen, die Auskunft über reale Einsatzszenarien von Lehrkräften und deren damit verbundene Intentionen geben. Dieses Desiderat konnte auch die vorliegende Studie nicht auflösen. Solche Szenarien sind schwierig zu erfassen, da mitunter eine hohe Zahl an Variablen ein solches Szenario beschreibt und bestimmt. Zwar wurden in dieser Erhebung für Modelle einige Rahmenbedingungen seitens der Schule und der Lehrkraft erhoben, diese können hier aber nicht kausal interpretiert werden. Mögliche Szenarien könnten an einer genaueren Untersuchung der Anwendung digitaler Medien zur Datenerfassung und -bearbeitung oder der Nutzung von Simulations‑, Experimentier- oder Modellbildungs‑/Modellierungsprogrammen ansetzen, da deren Einsatz im naturwissenschaftlichen Unterricht bisher als erweiterbar beschrieben wird (Kramer et al. 2019; Lorenz et al. 2017b).

Ein zumindest in Teilen überraschendes Desiderat ergibt sich aus der Nutzungszeit und Verfügbarkeit von digitalen Medien. Einerseits bewegt sich die Verfügbarkeit im Rahmen anderer Untersuchungen (Lorenz et al. 2017a; Pietzner 2009). Gleichwohl muss festgehalten werden, dass die Angaben aus der vorliegenden Untersuchung allenfalls auf eine Grundausstattung hindeuten, in dem Sinne, dass bestimmte Medien zumindest in geringer Stückzahl in den Fachräumen verfügbar sind. Dies ist insofern überraschend, als dass den Naturwissenschaften eine hohe Affinität zur Technik zugesprochen wird. Entsprechend gibt es auch viele Projekte an den Universitäten, in denen durchaus sehr konkrete Unterrichtsvorhaben entwickelt werden. Diese scheinen noch nicht in der Breite in der Unterrichtspraxis angekommen zu sein, auch aus Ausstattungsgründen. Aus Perspektive von Schulentwicklung wäre an diesem Punkt anzusetzen und es gälte Modelle für die Transformation der Ausstattung von Schulen mit Medien zu entwickeln. Neben der Ausstattung leuchtet zudem ein, dass die bloße Verfügbarkeit von Medien jedweder Form die Unterrichtsqualität nicht automatisch erhöht. Wie sich in der vorliegenden Befragung gezeigt hat, sind hier neben fachübergreifenden, mediendidaktischen Aspekten auch fachspezifische Aspekte verstärkt zu berücksichtigen. Welchen Anteil kann welches Fach zur Medienbildung spezifisch beitragen?

Geht es um konkrete Einsatzszenarien für Medien im Sinne der Heuristik gilt es basierend auf dieser Untersuchung, den Medieneinsatz neu zu bewerten. Ausgehend von der Frage „Was wirkt am besten?“ sollten fachdidaktisch und pädagogisch-psychologisch begründete Szenarien entwickelt und evaluiert werden. Dies ist sicher ein aktuelles und vielfach gefordertes Desiderat. Des Weiteren wäre in diesem Zusammenhang die daraus folgende Beschreibung und Analyse von Unterrichtsqualität unter Berücksichtigung des Medieneinsatzes wünschenswert. Einleitend waren die Merkmale von Medien und das sich daraus ergebende Potenzial für das Lehren und Lernen erläutert worden. Welche Schülergruppen profitieren von welchen Medien unter welchen Rahmenbedingungen am besten? Die Heuristik zur Beschreibung und Analyse von Merkmalen der Medien sowie ihres Einsatzes böte hier eine Basis, von der ausgehend gleichzeitig die dem Medieneinsatz zugeschriebenen Funktionen nicht außer Acht gelassen würden.

## Supplementary Information








## Footnotes

1 Die Befunde zu Mathematik sind ebenfalls veröffentlicht: Ostermann et al. 2021.

2 Diese Auswahl lässt sich kritisch diskutieren. An dieser Stelle ist es bedeutsam, über den größeren Rahmen des Projekts zu informieren: Das Gesamtprojekt umfasste nicht nur die drei Naturwissenschaften, sondern auch die Mathematik sowie außerschulische Lernorte. Ziel war es, alle vier – sofern möglich – miteinander zu vergleichen. Aus diesem Grund wurde für diese (und alle anderen) Frage(n) auch überlegt, welche Medien die Fächer teilen oder auch trennen. Bezogen auf digitale Medien entwickelte sich auch aufgrund von Gesprächen mit Expert*innen der Eindruck, dass im naturwissenschaftlichen Unterricht Hardware eine immer größere Rolle spielt, durch die eingebaute Messtechnik in Smartphones, während im Mathematikunterricht Software wie Computeralgebrasysteme häufig genutzt wird.
