# Supplementary material for: Is There a Subject Specific Use of Media in Science? Results of a Questionnaire Survey
Source: Z Didakt Nat Wiss. 2021 Jun 18;27(1):139–54. [Article in German] doi: 10.1007/s40573-021-00130-5 (PMC8212071; doi:10.1007/s40573-021-00130-5)
Supplement: Supplementary file 2 [file 40573_2021_130_MOESM2_ESM.docx]

**Fragebogenteil zum persönlichen Hintergrund**

Zu Beginn benötigen wir einige Informationen zu Ihrem beruflichen Hintergrund:

1. In welchem Bundesland unterrichten Sie?

| □ Baden-Württemberg | □ Niedersachsen |
| --- | --- |
| □ Bayern | □ Nordrhein-Westfalen |
| □ Berlin | □ Rheinland-Pfalz |
| □ Brandenburg | □ Saarland |
| □ Bremen | □ Sachsen |
| □ Hamburg | □ Sachsen-Anhalt |
| □ Hessen | □ Schleswig-Holstein |
| □ Mecklenburg-Vorpommern | □ Thüringen |

1. Welchem Bildungsgang bzw. welchen Bildungsgängen lässt sich die Schulform, an der Sie unterrichten, am ehesten zuordnen?

*(Hinweis: Die Nennung der Bildungsgänge orientiert sich an der „Grundstruktur des Bildungswesen in der Bundesrepublik Deutschland“, wie sie von der Kultusministerkonferenz beschrieben wird.)*

| □ | Schulart nur mit Hauptschulbildungsgang |
| --- | --- |
| □ | Schulart nur mit Realschulbildungsgang |
| □ | Schulart nur mit gymnasialem Bildungsgang |
| □ | Schulart mit Hauptschul- und Realschulbildungsgang |
| □ | Schulart mit Hauptschul-, Realschul- und gymnasialen Bildungsgang ohne gymnasiale Oberstufe |
| □ | Schulart mit Hauptschul-, Realschul- und gymnasialen Bildungsgang mit gymnasialer Oberstufe |

1. In welchen Schulstufen unterrichten Sie?

| □ Sekundarstufe I |
| --- |
| □ Sekundarstufe II |
| □ Sonstiges, und zwar:______________________________ |

1. Welche MINT-Fächer unterrichten Sie?

| □ Mathematik |
| --- |
| □ Informatik |
| □ Biologie |
| □ Chemie |
| □ Physik |
| □ Naturwissenschaften integriert |
| □ Technik |
| □ _________________________ |

1. Seit wie vielen Jahren (inklusive Referendariat/Vorbereitungsdienst) unterrichten Sie?

| □ 0 – 2 Jahre |
| --- |
| □ 3 – 5 Jahre |
| □ 6 – 10 Jahre |
| □ 11 – 20 Jahre |
| □ Mehr als 20 Jahre |

1. Welches Geschlecht haben Sie?

| □ männlich |
| --- |
| □ weiblich |

1. Wo haben Sie Ihr medienbezogenes Wissen/Ihre medienbezogenen Kompetenzen erworben? Bitte geben Sie den jeweiligen Anteil bezogen auf 100 % an.

| _____% | im Studium |
| --- | --- |
| _____% | im Referendariat |
| _____% | in berufsbegleitenden Weiterbildungen/Fortbildungen |
| _____% | durch kollegialen Austausch |
| _____% | im Selbststudium |

1. Haben Sie in den Schuljahren 2015/16 oder 2016/17 Fortbildungen zur Nutzung von Medien im Unterricht besucht?

| □ Ja |
| --- |
| □ Nein |

**Fragebogenteil zu außerschulischen Lernorten**

Haben Sie in den Schuljahren 2015/16 oder 2016/17 außerschulische Lernorte besucht?

| □ Ja | □ Nein |
| --- | --- |

Wenn Sie in den Schuljahren 2015/16 oder 2016/17 **keine außerschulischen Lernorte** besucht haben, fahren Sie bitte auf S. 7 fort.

Stellen Sie sich für die Beantwortung der folgenden Fragen bitte zwei konkrete Besuche vor, die Sie in den Schuljahren 2015/16 oder 2016/17 gemacht haben. Sollten Sie nur einen Besuch gemacht haben, lassen Sie bitte die zweite Spalte frei.

|  | Lernort 1 | | Lernort 2 | |
| --- | --- | --- | --- | --- |
| Hier war ich mit einer Klasse des | ______. Jahrgangs | | ______. Jahrgangs | |
| Welche Lernorte haben Sie besucht? | | | | |
| - Museum/Ausstellung/Science Center | □ | | □ | |
| - Schülerlabor (z. B. an einer Uni, in einem Museum, Unternehmen) | □ | | □ | |
| - Industriebetriebe (z.B. Produktion), Forschungsgruppen an Universitäten o. ä. | □ | | □ | |
| - Andere, und zwar … | □_______________________ | | □_______________________ | |
| Wie viel Zeit haben Sie in die Vorbereitung des Besuchs investiert? | | | | |
| - Eigene Vorbereitung/Planung (Lehrkraft): |  | |  | |
| - Organisatorisch | ______________________min | | ______________________min | |
| - Fachlich/inhaltlich | ______________________min | | ______________________min | |
| - Vorbereitung mit/in der Klasse: |  | |  | |
| - Organisatorisch | ______________________min | | ______________________min | |
| - Fachlich/inhaltlich | ______________________min | | ______________________min | |
| - Vorbereitung fand im Wesentlichen am außerschulischen Lernort statt. | ______________________min | | ______________________min | |
| Haben Sie zum Besuch des außerschulischen Lernorts etwas mitgebracht? | | | | |
| (z. B. Fragenkatalog der Schülerinnen und Schüler, Material zur Untersuchung im Labor, …) | □ Nein | | □ Nein | |
|  | □ Ja, folgendes:  ___________________________________________________________________________ | | □ Ja, folgendes:  ___________________________________________________________________________ | |
| Wie viel Zeit haben Sie in die Nachbereitung des Besuchs investiert? | | | | |
| - Nachbereitung im Unterricht | ______________________min | | ______________________min | |
| - Nachbereitung individuell (z. B. zu Hause) | ______________________min | | ______________________min | |
| - Nachbereitung am außerschulischen Lernort | ______________________min | | ______________________min | |
| Welche Formate haben Sie für die Nachbereitung genutzt? | | | | |
| - Keine unmittelbare Nachbereitung | □ | | □ | |
| - Unterrichtsgespräch | □ | | □ | |
| - Schülerpräsentation | □ | | □ | |
| - Bericht in Jahres-/Schulbericht, Homepage | □ | | □ | |
| - Andere | ____________________________________________________________________________________________________ | | ____________________________________________________________________________________________________ | |
|  | Lernort 1 | | Lernort 2 | |
| Um die Nachbereitung des Besuchs zu vereinfachen gibt es je nach Lernort verschiedene Medien und Angebote.  Welche Angebote wurden Ihnen angeboten, welche haben Sie genutzt? | Angeboten | Genutzt | Angeboten | Genutzt |
| - Allgemeines Infomaterial/Broschüren | □ | □ | □ | □ |
| - Spezielles Infomaterial für Lehrkräfte | □ | □ | □ | □ |
| - Arbeitsblätter/Arbeitsmappen für Schülerinnen und Schüler | □ | □ | □ | □ |
| - Dokumentation durch Fotos (selbst/durch Schülerinnen und Schüler gemachte) | □ | □ | □ | □ |
| - Messdaten oder andere digitale Arbeitsprodukte (z. B. auf USB-Stick, per E-Mail) | □ | □ | □ | □ |
| - Reale Objekte (z. B. Produkte des Schülerlaborbesuchs, Anschauungsmaterial aus einem Naturkundemuseum, …) | □ | □ | □ | □ |
| - Andere | ____________________________________ | ______________________________ | ______________________________ | ____________________________________ |

**Fragebogenteil für Lehrkräfte mit dem Fach Chemie**

1. **Medieneinsatz im Chemieunterricht**
2. Für die Gestaltung von Chemieunterricht in der 8. und 9. Jahrgangsstufe können unterschiedliche Medien eingesetzt werden. Bitte geben Sie für die nachfolgend genannten Medien die von Ihnen geschätzte Nutzungsdauer für Ihren Chemieunterricht an. Stellen Sie sich dabei eine typische, von Ihnen geplante Unterrichtseinheit zum Thema Säuren und Basen vor. **Schätzen Sie ab**, wie lange Sie in Ihrem Chemieunterricht das jeweilige Medium nutzen, wenn Sie ein Inhaltsgebiet über vier Wochen hinweg unterrichten (zwei Unterrichtsstunden pro Woche, 360 Minuten in vier Wochen).

*Bitte geben Sie die* ***geschätzte Zeit*** *in Minuten in der folgenden Tabelle an. Sie haben die Möglichkeit, weitere Medien zu ergänzen.*

| Von den ca. 360 Minuten einer durchschnittlichen Unterrichtseinheit nutzen die Schülerinnen und Schüler … | |
| --- | --- |
| ein gegenständliches Modell | ca. _____ Minuten. |
| eine virtuelle Lernumgebung | ca. _____ Minuten. |
| ein reales Experiment | ca. _____ Minuten. |
| ein Smartphone | ca. _____ Minuten. |
| einen Tablet-PC | ca. _____ Minuten. |
| ein Notebook | ca. _____ Minuten. |
| einen Computer/Desktop PC | ca. _____ Minuten. |
| einen Overhead-Projektor | ca. _____ Minuten. |
| eine interaktive Tafel | ca. _____ Minuten. |
| ein Schulbuch | ca. _____ Minuten. |
| _________________________ | ca. _____ Minuten. |
| _________________________ | ca. _____ Minuten. |

1. Haben Sie in den letzten 5 Jahren die Jahrgangsstufe 8 oder 9 zum Thema Säuren und Basen unterrichtet?
   □ Ja □ Nein
2. **Modelle und Modellexperimente im Chemieunterricht**


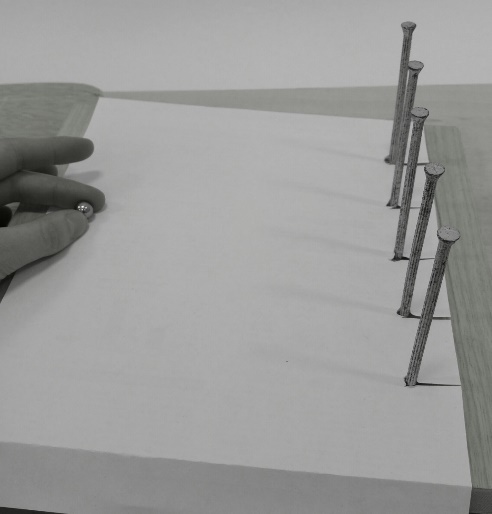
In der Chemie gibt es eine Reihe von Modellexperimenten. Abbildung 1 zeigt den Aufbau eines solchen Modellexperiments, den Aufbau des Rutherford‘schen Streuversuchs. Anhand dieses beispielhaft dargestellten Modellexperiments können Schlussfolgerungen über den Aufbau der Atome gezogen werden. Schülerinnen und Schüler können in diesem Zusammenhang ein Modellexperiment selbst durchführen. Bei diesem lassen die Schülerinnen und Schüler eine Kugel (α-Strahlen) 50-mal eine schräg aufgestellte Platte herunterrollen. Auf der Platte sind auf einer Höhe Nägel (Atomkerne) befestigt. Die Lernenden notieren jedes Mal, ob die Kugel einen Nagel genau trifft, ihn streift oder zwischen den Nägeln ohne Berührung hindurch läuft.

Abbildung 1: Beispiel für ein Modellexperiment zum Rutherfordschen Streuversuch

1. Modelle und Modellexperimente für den Chemieunterricht sind an meiner Schule …

| □ für alle Lernenden immer verfügbar. |
| --- |
| □ für alle Lernenden bei Bedarf als mobiler Klassensatz verfügbar. |
| □ für alle Lernenden bei Bedarf im Chemieraum verfügbar. |
| □ in Form einzelner Aufbauten bei Bedarf verfügbar |
| □ nicht verfügbar. |

Wenn an Ihrer Schule **keine Modelle und Modellexperimente für den Chemieunterricht vorhanden** sind, fahren Sie bitte mit Abschnitt C (Virtuelle Lernumgebungen im Chemieunterricht) auf S. 9 fort.

1. Wie oft führen Ihre Schülerinnen und Schüler folgende Lernaktivitäten bei der Nutzung von Modellexperimenten im Chemieunterricht typischerweise durch? Denken Sie dabei an **eine konkrete** Klasse, die Sie in Chemie unterrichten.

| Meine Schülerinnen und Schüler nutzen Modelle oder Modellexperimente im Chemieunterricht … | in keiner oder fast keiner Unterrichtsstunde | in weniger als der Hälfte der Unterrichtsstunden | in mindestens der Hälfte der Unterrichtsstunden | in jeder oder fast jeder Unterrichtsstunde |
| --- | --- | --- | --- | --- |
| zur modellhaften Beschreibung des Aufbaus von Stoffen und Atomen. | □ | □ | □ | □ |
| zur Deutung von Stoffeigenschaften auf Teilchenebene. | □ | □ | □ | □ |
| zur Deutung von Stoff- und Energieumwandlungen hinsichtlich der Veränderung von Teilchen und des Umbaus chemischer Bindungen. | □ | □ | □ | □ |
| zum Durchführen von qualitativen und quantitativen Untersuchungen und zum Überprüfen von Hypothesen. | □ | □ | □ | □ |
| zum Erheben von Daten. | □ | □ | □ | □ |
| zum Herausfinden von Trends und Zusammenhängen. | □ | □ | □ | □ |
| zum Analysieren von Strukturen und Beziehungen in Daten. | □ | □ | □ | □ |
| zur Beantwortung chemischer Fragestellungen. | □ | □ | □ | □ |
| zum Veranschaulichen und Erklären chemischer Sachverhalte. | □ | □ | □ | □ |
| zum Reflektieren, in welchem Verhältnis Modell und Wirklichkeit zueinander stehen. | □ | □ | □ | □ |
| zum Bewerten der Grenzen des Modells. | □ | □ | □ | □ |
|  |  |  |  |  |
| *Hier haben Sie die Möglichkeit weitere Aktivitäten zu ergänzen:* |  |  |  |  |
|  | □ | □ | □ | □ |
|  | □ | □ | □ | □ |

1. Alles in allem: Wie zufrieden sind Sie mit der derzeitigen Ausstattung mit Modellen und Modellexperimenten für den Chemieunterricht an Ihrer Schule?

| sehr unzufrieden | eher unzufrieden | eher zufrieden | sehr zufrieden |
| --- | --- | --- | --- |
| □ | □ | □ | □ |

1. **Virtuelle Lernumgebungen im Chemieunterricht**

Durch die Nutzung neuer Medien lassen sich viele Experimente aus dem Chemieunterricht in virtueller Form durchführen. Ein Beispiel für eine virtuelle Lernumgebung ist das Virtual Chemistry Lab, welches unter anderem die Möglichkeit bietet, eine Säure-Base-Titration durchzuführen (s. Abbildung 2). In einem virtuellen Sammlungsraum stehen verschiedene Chemikalien und Geräte zum Experimentieren zur Verfügung, wie etwa 1 M Chlorwasserstofflösung, 1 M Natriumhydroxidlösung und Phenolphthaleinlösung. Außerdem gibt es ein Feld mit Informationen zum Namen und Volumen der Lösung, zu den enthaltenen Teilchen, zur Temperatur und zum pH-Wert.


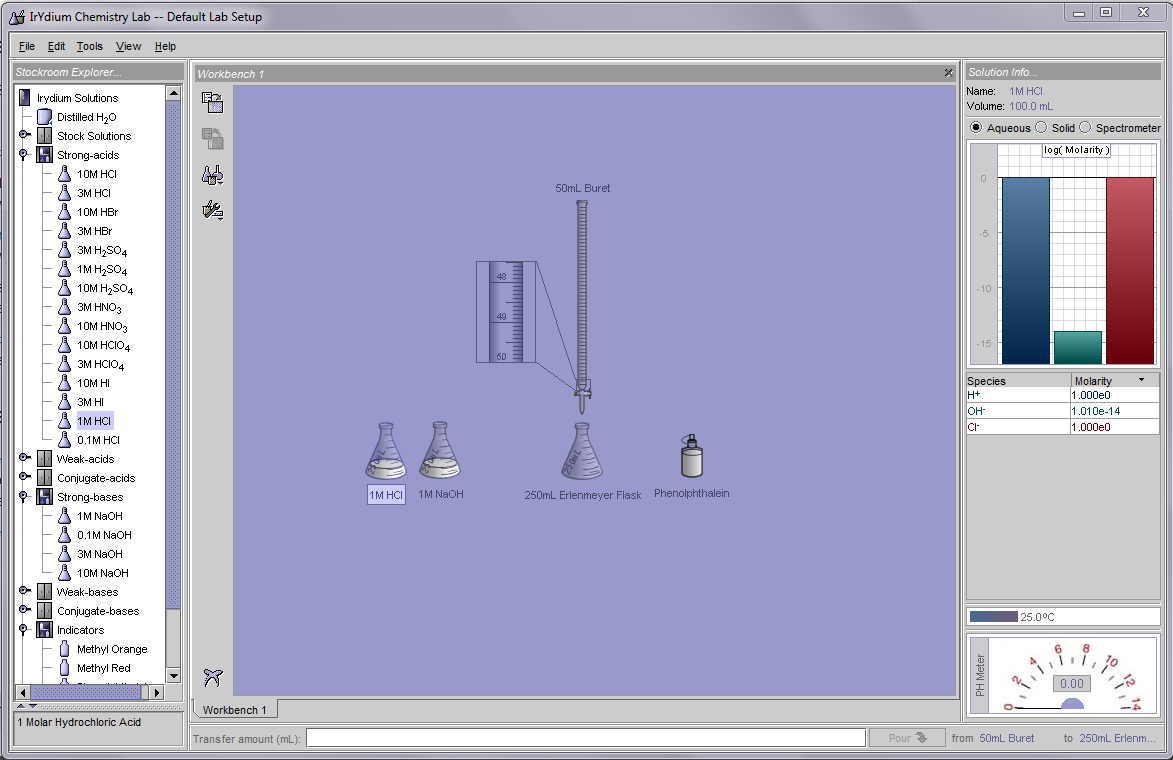


Abbildung 2: Beispiel für einen virtuellen Lernraum zum Experimentieren, das Virtual Chemistry Lab.

1. Virtuelle Lernumgebungen können im Chemieunterricht an meiner Schule …

| □ durch alle Lernenden immer genutzt werden. |
| --- |
| □ durch alle Lernenden bei Bedarf im Klassensatz genutzt werden. |
| □ durch alle Lernenden bei Bedarf im Computerraum genutzt werden. |
| □ durch einzelne Lernende bei Bedarf in Form einzelner Lizenzen genutzt werden. |
| □ nicht genutzt werden. |

Wenn an Ihrer Schule **keine virtuellen Lernumgebungen für den Chemieunterricht** vorhanden sind, fahren Sie bitte mit Abschnitt D (Bekanntheitsgrad der Mediennutzung) auf S. 10 fort.

1. Wie oft führen Ihre Schülerinnen und Schüler folgende Lernaktivitäten bei der Nutzung von virtuellen Lernumgebungen im Chemieunterricht typischerweise durch? Denken Sie dabei an **eine konkrete** Klasse, die Sie in Chemie unterrichten.

| Meine Schülerinnen und Schüler nutzen virtuelle Lernumgebungen im Chemieunterricht … | in keiner oder fast keiner Unterrichtsstunde | in weniger als der Hälfte der Unterrichtsstunden | in mindestens der Hälfte der Unterrichtsstunden | in jeder oder fast jeder Unterrichtsstunde |
| --- | --- | --- | --- | --- |
| zur modellhaften Beschreibung des Aufbaus von Stoffen und Atomen. | □ | □ | □ | □ |
| zur Deutung von Stoffeigenschaften auf Teilchenebene. | □ | □ | □ | □ |
| zur Deutung von Stoff- und Energieumwandlungen hinsichtlich der Veränderung von Teilchen und des Umbaus chemischer Bindungen. | □ | □ | □ | □ |
| zum Entwickeln von Fragestellungen. | □ | □ | □ | □ |
| zum Durchführen von qualitativen und quantitativen Untersuchungen und zum Überprüfen von Hypothesen. | □ | □ | □ | □ |
| zum Erheben von Daten. | □ | □ | □ | □ |
| zum Herausfinden von Trends oder Zusammenhängen. | □ | □ | □ | □ |
| zum Lernen der Bedienung der virtuellen Lernumgebung. | □ | □ | □ | □ |
| zum Analysieren von Strukturen und Beziehungen in Daten. | □ | □ | □ | □ |
| zum Beschreiben und Erklären chemischer Sachverhalte. | □ | □ | □ | □ |
| zur Veranschaulichung chemischer Sachverhalte anhand von Modellen und Darstellungen. | □ | □ | □ | □ |
| zum Protokollieren des Verlaufs und der Ergebnisse von Untersuchungen und Diskussionen. | □ | □ | □ | □ |
|  |  |  |  |  |
| *Hier haben Sie die Möglichkeit weitere Aktivitäten zu ergänzen:* |  |  |  |  |
|  | □ | □ | □ | □ |
|  | □ | □ | □ | □ |

1. Alles in allem: Wie zufrieden sind Sie mit der derzeitigen Verfügbarkeit virtueller Lernumgebungen für den Chemieunterricht an Ihrer Schule?

| sehr unzufrieden | eher unzufrieden | eher zufrieden | sehr zufrieden |
| --- | --- | --- | --- |
| □ | □ | □ | □ |

1. **Bekanntheitsgrad der Mediennutzung**

Gleich geschafft! Nach dem fachlichen Teil haben wir noch ein paar Fragen, um besser einschätzen zu können, welche Bedürfnisse Sie als Lehrkraft in Bezug auf mediengestütztes Unterrichten haben.

Im Folgenden finden Sie verschiedene Einstellungen, Haltungen und Positionen, die Sie als Lehrkraft während der gedanklichen Beschäftigung mit dem mediengestützten Unterrichten bzw. während der Umsetzung mediengestützten Unterrichtens haben können. Es ist möglich, dass Sie bereits mit dem mediengestützten Unterrichten mit Modellexperimenten und/oder virtuellen Lernumgebungen vertraut sind oder dass Ihnen dieses Unterrichten wenig geläufig ist. Es kann auch sein, dass die Inhalte mancher Aussagen für Sie zurzeit gar keine Relevanz besitzen. Gehen Sie daher bei der Beantwortung folgendermaßen vor:

Kreuzen Sie bitte an, wie stark die Aussage auf **Sie persönlich** zum **jetzigen Zeitpunkt** zutrifft. Hierfür stehen Ihnen sieben Abstufungen (1 bis 7) zur Verfügung. Wenn Sie aber gar nichts mit der Aussage anfangen können (z. B. weil Sie sich noch nie Gedanken dazu gemacht haben), dann kreuzen Sie bitte das Kästchen „zurzeit nicht relevant“ (0) an. Bitte beantworten Sie die folgenden Aussagen nach der eben beschriebenen Vorgehensweise.

|  | Medium: Modelle und Modellexperimente | | | | | | | |  | Medium: virtuelle Lernumgebungen | | | | | | | |
| --- | --- | --- | --- | --- | --- | --- | --- | --- | --- | --- | --- | --- | --- | --- | --- | --- | --- |
|  | 0 zurzeit nicht relevant | 1 trifft zurzeit gar nicht auf mich zu | 2 | 3 | 4 | 5 | 6 | 7 trifft zurzeit völlig auf mich zu |  | 0 zurzeit nicht relevant | 1 trifft zurzeit gar nicht auf mich zu | 2 | 3 | 4 | 5 | 6 | 7 trifft zurzeit völlig auf mich zu |
| Ich würde mich gerne darüber austauschen, wie man mathematisch-naturwissenschaftlichen Unterricht mediengestützt gestalten kann. | □ | □ | □ | □ | □ | □ | □ | □ |  | □ | □ | □ | □ | □ | □ | □ | □ |
| Ich wüsste gerne, welche Ressourcen dafür zur Verfügung stehen. | □ | □ | □ | □ | □ | □ | □ | □ |  | □ | □ | □ | □ | □ | □ | □ | □ |
| Ich will wissen, inwiefern das Unterrichten mit dem Medium besser ist. | □ | □ | □ | □ | □ | □ | □ | □ |  | □ | □ | □ | □ | □ | □ | □ | □ |
| Ich würde gerne wissen, wie sich meine Aufgaben beim Unterrichten mit Medien konkret verändern sollen. | □ | □ | □ | □ | □ | □ | □ | □ |  | □ | □ | □ | □ | □ | □ | □ | □ |
| Ich hätte gerne mehr Informationen über den nötigen Arbeits- und Zeitaufwand beim Unterrichten mit Medien. | □ | □ | □ | □ | □ | □ | □ | □ |  | □ | □ | □ | □ | □ | □ | □ | □ |
| Ich möchte gerne wissen, wie sich meine Rolle durch den Einsatz des Mediums verändert. | □ | □ | □ | □ | □ | □ | □ | □ |  | □ | □ | □ | □ | □ | □ | □ | □ |
| Ich mache mir Gedanken über die Auswirkungen von mediengestütztem Unterrichten auf die Schülerinnen und Schüler. | □ | □ | □ | □ | □ | □ | □ | □ |  | □ | □ | □ | □ | □ | □ | □ | □ |
| Ich überlege wie ich die Wirkung der Medien auf die Schülerinnen und Schüler überprüfen kann. | □ | □ | □ | □ | □ | □ | □ | □ |  | □ | □ | □ | □ | □ | □ | □ | □ |
| Ich möchte Schülerrückmeldungen dazu nutzen, um die Verwendung von Medien fortzuentwickeln. | □ | □ | □ | □ | □ | □ | □ | □ |  | □ | □ | □ | □ | □ | □ | □ | □ |
| Ich würde gerne mit anderen Lehrkräften zum unterrichtspraktischen Einsatz von Medien zusammenarbeiten. | □ | □ | □ | □ | □ | □ | □ | □ |  | □ | □ | □ | □ | □ | □ | □ | □ |
| Ich möchte gerne meine Aktivitäten mit anderen koordinieren, um die positive Wirkung des Medieneinsatzes im Unterricht zu maximieren. | □ | □ | □ | □ | □ | □ | □ | □ |  | □ | □ | □ | □ | □ | □ | □ | □ |
| Mich interessiert, was andere Lehrkräfte auf dem Gebiet des mediengestützten Unterrichts unternehmen. | □ | □ | □ | □ | □ | □ | □ | □ |  | □ | □ | □ | □ | □ | □ | □ | □ |

1. **Desktop-Computer und Mobile-Computer im Chemieunterricht**

Für die Durchführung von Chemieunterricht wird aktuell die Nutzung digitaler Medien diskutiert. Zunehmend stehen in Schulen digitale Medien wie Desktop-Computer oder mobile Computer zur Verfügung.

1. Desktop-Computer oder mobile Lösungen wie Notebooks oder Tablets sind an meiner Schule zur Nutzung im Chemieunterricht …

| □ für alle Lernenden immer verfügbar. |
| --- |
| □ für alle Lernenden bei Bedarf als mobiler Klassensatz verfügbar. |
| □ für alle Lernenden bei Bedarf im Computerraum verfügbar. |
| □ in Form einzelner Geräte bei Bedarf verfügbar |
| □ nicht verfügbar. |

1. Wie oft führen Ihre Schülerinnen und Schüler die nachfolgend aufgeführten Lernaktivitäten im Chemieunterricht mit Desktop-Computern oder mobilen Computern durch? Denken Sie dabei an **eine konkrete** Klasse, die Sie in Chemie unterrichten.

| Meine Schülerinnen und Schüler nutzen Computer, Tablets und/oder Smartphones im Chemieunterricht … | in keiner oder fast keiner Unterrichtsstunde | in weniger als der Hälfte der Unterrichtsstunden | in mindestens der Hälfte der Unterrichtsstunden | in jeder oder fast jeder Unterrichtsstunde |
| --- | --- | --- | --- | --- |
| zum Erstellen von Präsentationen oder zum Schreiben eines Textes. | □ | □ | □ | □ |
| zum Recherchieren von Informationen. | □ | □ | □ | □ |
| zum Zeichnen von Molekülstrukturen oder Versuchsaufbauten. | □ | □ | □ | □ |
| zum Kommunizieren mit Mitschülerinnen und Mitschülern. | □ | □ | □ | □ |
| zum Überprüfen des aktuellen Leistungsstandes. | □ | □ | □ | □ |
| zum Üben und Trainieren. | □ | □ | □ | □ |
| zum Simulieren oder zum Modellieren fachlicher Prozesse. | □ | □ | □ | □ |
| zum Messen von z. B. pH-Wert oder Temperatur. | □ | □ | □ | □ |
| zum Bearbeiten von Filmen und Bildern. | □ | □ | □ | □ |
|  |  |  |  |  |
| *Hier haben Sie die Möglichkeit weitere Aktivitäten zu ergänzen:* |  |  |  |  |
|  | □ | □ | □ | □ |
|  | □ | □ | □ | □ |

1. Alles in allem: Wie zufrieden sind Sie mit der derzeitigen Ausstattung mit Desktop Computern oder mobilen Lösungen an Ihrer Schule?

| sehr unzufrieden | eher unzufrieden | eher zufrieden | sehr zufrieden |
| --- | --- | --- | --- |
| □ | □ | □ | □ |

1. **Einstellungen zum Medieneinsatz allgemein**
2. Im Folgenden finden Sie verschiedene Aussagen zu digitalen Medien im Unterricht. Geben Sie bitte an, inwiefern Sie den Aussagen zustimmen. Es gibt dabei keine richtigen oder falschen Antworten. Bitte setzen Sie je Aussage nur ein Kreuz, das auf Ihren Standpunkt am besten zutrifft.

|  | stimme überhaupt nicht zu | stimme eher nicht zu | neutral | stimme eher zu | stimme voll zu |
| --- | --- | --- | --- | --- | --- |
| Digitale Medien im Unterricht haben nur Unterhaltungswert für Schülerinnen und Schüler. | □ | □ | □ | □ | □ |
| Digitale Medien können den Lernerfolg bei Schülerinnen und Schülern positiv beeinflussen. | □ | □ | □ | □ | □ |
| Digitale Medien verschlechtern die Qualität des Unterrichts. | □ | □ | □ | □ | □ |
| Digitale Medien unterstützen Schülerinnen und Schüler beim Lernen. | □ | □ | □ | □ | □ |
| Digitale Medien sorgen im Unterricht für Unruhe und lenken vom Lerninhalt ab. | □ | □ | □ | □ | □ |
| Der Einsatz von digitalen Medien macht den Unterricht effektiver und effizienter. | □ | □ | □ | □ | □ |

1. Wie würden Sie Ihr Verhältnis zu digitalen Medien generell beschreiben?

| Ich kann mit digitalen Medien nichts anfangen | Ich stehe digitalen Medien eher skeptisch gegenüber | Ich stehe digitalen Medien eher positiv gegenüber | Ich bin ein Technik-Fan |
| --- | --- | --- | --- |
| □ | □ | □ | □ |

1. Wie stehen Sie dem Einsatz digitaler Medien im Unterricht im Großen und Ganzen gegenüber?

| negativ | eher negativ | eher positiv | positiv |
| --- | --- | --- | --- |
| □ | □ | □ | □ |

1. Bitte bewerten Sie, inwiefern Sie den folgenden Aussagen zustimmen.

|  | stimmt nicht | stimmt kaum | stimmt eher | stimmt genau |
| --- | --- | --- | --- | --- |
| Ich kann in meiner Unterrichtsplanung zu den Lernzielen passende Einsätze digitaler Medien planen, auch wenn meine Schule nicht optimal mit digitalen Medien ausgestattet ist. | □ | □ | □ | □ |
| Ich kann den Einsatz digitaler Medien im Fachunterricht so planen, dass meine Schülerinnen und Schüler begeistert sind, auch wenn sie sich sonst wenig für das Fach interessieren. | □ | □ | □ | □ |
| Ich kann den Einsatz eines digitalen Mediums im Fachunterricht didaktisch begründen, auch wenn ich dieses digitale Medium noch nicht selbst eingesetzt habe. | □ | □ | □ | □ |
| Ich kann eine Lösung für technische Probleme beim Unterrichten mit digitalen Medien finden, auch wenn ich unter Zeitdruck stehe. | □ | □ | □ | □ |
| Ich kann den Einsatz digitaler Medien so gestalten, dass meine Schülerinnen und Schüler motiviert sind mitzuarbeiten, auch wenn es sich um eine unbeliebte Randstunde handelt. | □ | □ | □ | □ |
| Ich kann den fachlichen Lernprozess durch den Einsatz digitaler Medien unterstützen, auch wenn unvorhergesehene Verständnisschwierigkeiten auftreten. | □ | □ | □ | □ |

1. Was ist Ihrer Meinung nach die größte Herausforderung für den Unterricht mit digitalen Medien?

|  |
| --- |

1. Haben Sie Anmerkungen zum Fragebogen? Geben Sie uns Feedback, damit wir den Fragebogen besser gestalten können!

|  |
| --- |

**Vielen Dank für Ihre Unterstützung!**
