# Supplementary material for: Is There a Subject Specific Use of Media in Science? Results of a Questionnaire Survey
Source: Z Didakt Nat Wiss. 2021 Jun 18;27(1):139–54. [Article in German] doi: 10.1007/s40573-021-00130-5 (PMC8212071; doi:10.1007/s40573-021-00130-5)
Supplement: Supplementary file 3 [file 40573_2021_130_MOESM3_ESM.docx]

**Fragebogenteil zum persönlichen Hintergrund**

Zu Beginn benötigen wir einige Informationen zu Ihrem beruflichen Hintergrund:

1. In welchem Bundesland unterrichten Sie?

| □ Baden-Württemberg | □ Niedersachsen |
| --- | --- |
| □ Bayern | □ Nordrhein-Westfalen |
| □ Berlin | □ Rheinland-Pfalz |
| □ Brandenburg | □ Saarland |
| □ Bremen | □ Sachsen |
| □ Hamburg | □ Sachsen-Anhalt |
| □ Hessen | □ Schleswig-Holstein |
| □ Mecklenburg-Vorpommern | □ Thüringen |

1. Welchem Bildungsgang bzw. welchen Bildungsgängen lässt sich die Schulform, an der Sie unterrichten, am ehesten zuordnen?

*(Hinweis: Die Nennung der Bildungsgänge orientiert sich an der „Grundstruktur des Bildungswesen in der Bundesrepublik Deutschland“, wie sie von der Kultusministerkonferenz beschrieben wird.)*

| □ | Schulart nur mit Hauptschulbildungsgang |
| --- | --- |
| □ | Schulart nur mit Realschulbildungsgang |
| □ | Schulart nur mit gymnasialem Bildungsgang |
| □ | Schulart mit Hauptschul- und Realschulbildungsgang |
| □ | Schulart mit Hauptschul-, Realschul- und gymnasialen Bildungsgang ohne gymnasiale Oberstufe |
| □ | Schulart mit Hauptschul-, Realschul- und gymnasialen Bildungsgang mit gymnasialer Oberstufe |

1. In welchen Schulstufen unterrichten Sie?

| □ Sekundarstufe I |
| --- |
| □ Sekundarstufe II |
| □ Sonstiges, und zwar:______________________________ |

1. Welche MINT-Fächer unterrichten Sie?

| □ Mathematik |
| --- |
| □ Informatik |
| □ Biologie |
| □ Chemie |
| □ Physik |
| □ Naturwissenschaften integriert |
| □ Technik |
| □ _________________________ |

1. Seit wie vielen Jahren (inklusive Referendariat/Vorbereitungsdienst) unterrichten Sie?

| □ 0 – 2 Jahre |
| --- |
| □ 3 – 5 Jahre |
| □ 6 – 10 Jahre |
| □ 11 – 20 Jahre |
| □ Mehr als 20 Jahre |

1. Welches Geschlecht haben Sie?

| □ männlich |
| --- |
| □ weiblich |

1. Wo haben Sie Ihr medienbezogenes Wissen/Ihre medienbezogenen Kompetenzen erworben? Bitte geben Sie den jeweiligen Anteil bezogen auf 100 % an.

| _____% | im Studium |
| --- | --- |
| _____% | im Referendariat |
| _____% | in berufsbegleitenden Weiterbildungen/Fortbildungen |
| _____% | durch kollegialen Austausch |
| _____% | im Selbststudium |

1. Haben Sie in den Schuljahren 2015/16 oder 2016/17 Fortbildungen zur Nutzung von Medien im Unterricht besucht?

| □ Ja |
| --- |
| □ Nein |

**Fragebogenteil zu außerschulischen Lernorten**

Haben Sie in den Schuljahren 2015/16 oder 2016/17 außerschulische Lernorte besucht?

| □ Ja | □ Nein |
| --- | --- |

Wenn Sie in den Schuljahren 2015/16 oder 2016/17 **keine außerschulischen Lernorte besucht** haben, fahren Sie bitte auf S. 7 fort.

Stellen Sie sich für die Beantwortung der folgenden Fragen bitte zwei konkrete Besuche vor, die Sie in den Schuljahren 2015/16 oder 2016/17 gemacht haben. Sollten Sie nur einen Besuch gemacht haben, lassen Sie bitte die zweite Spalte frei.

|  | Lernort 1 | | Lernort 2 | |
| --- | --- | --- | --- | --- |
| Hier war ich mit einer Klasse des | ______. Jahrgangs | | ______. Jahrgangs | |
| Welche Lernorte haben Sie besucht? | | | | |
| - Museum/Ausstellung/Science Center | □ | | □ | |
| - Schülerlabor (z. B. an einer Uni, in einem Museum, Unternehmen) | □ | | □ | |
| - Industriebetriebe (z.B. Produktion), Forschungsgruppen an Universitäten o. ä. | □ | | □ | |
| - Andere, und zwar … | □_______________________ | | □_______________________ | |
| Wie viel Zeit haben Sie in die Vorbereitung des Besuchs investiert? | | | | |
| - Eigene Vorbereitung/Planung (Lehrkraft): |  | |  | |
| - Organisatorisch | ______________________min | | ______________________min | |
| - Fachlich/inhaltlich | ______________________min | | ______________________min | |
| - Vorbereitung mit/in der Klasse: |  | |  | |
| - Organisatorisch | ______________________min | | ______________________min | |
| - Fachlich/inhaltlich | ______________________min | | ______________________min | |
| - Vorbereitung fand im Wesentlichen am außerschulischen Lernort statt. | ______________________min | | ______________________min | |
| Haben Sie zum Besuch des außerschulischen Lernorts etwas mitgebracht? | | | | |
| (z. B. Fragenkatalog der Schülerinnen und Schüler, Material zur Untersuchung im Labor, …) | □ Nein | | □ Nein | |
|  | □ Ja, folgendes:  ___________________________________________________________________________ | | □ Ja, folgendes:  ___________________________________________________________________________ | |
| Wie viel Zeit haben Sie in die Nachbereitung des Besuchs investiert? | | | | |
| - Nachbereitung im Unterricht | ______________________min | | ______________________min | |
| - Nachbereitung individuell (z. B. zu Hause) | ______________________min | | ______________________min | |
| - Nachbereitung am außerschulischen Lernort | ______________________min | | ______________________min | |
| Welche Formate haben Sie für die Nachbereitung genutzt? | | | | |
| - Keine unmittelbare Nachbereitung | □ | | □ | |
| - Unterrichtsgespräch | □ | | □ | |
| - Schülerpräsentation | □ | | □ | |
| - Bericht in Jahres-/Schulbericht, Homepage | □ | | □ | |
| - Andere | _____________________________________________________________________________________________________________________________ | | _____________________________________________________________________________________________________________________________ | |
|  | Lernort 1 | | Lernort 2 | |
| Um die Nachbereitung des Besuchs zu vereinfachen gibt es je nach Lernort verschiedene Medien und Angebote.  Welche Angebote wurden Ihnen angeboten, welche haben Sie genutzt? | Angeboten | Genutzt | Angeboten | Genutzt |
| - Allgemeines Infomaterial/Broschüren | □ | □ | □ | □ |
| - Spezielles Infomaterial für Lehrkräfte | □ | □ | □ | □ |
| - Arbeitsblätter/Arbeitsmappen für Schülerinnen und Schüler | □ | □ | □ | □ |
| - Dokumentation durch Fotos (selbst/durch Schülerinnen und Schüler gemachte) | □ | □ | □ | □ |
| - Messdaten oder andere digitale Arbeitsprodukte (z. B. auf USB-Stick, per E-Mail) | □ | □ | □ | □ |
| - Reale Objekte (z. B. Produkte des Schülerlaborbesuchs, Anschauungsmaterial aus einem Naturkundemuseum, …) | □ | □ | □ | □ |
| - Andere | ____________________________________ | ______________________________ | ______________________________ | ____________________________________ |

Fragebogenteil für Lehrkräfte mit dem Fach Physik

1. Medieneinsatz im Physikunterricht
   1. Für die Gestaltung von Physikunterricht in der 8. und 9. Jahrgangsstufe können unterschiedliche Medien eingesetzt werden. Bitte geben Sie für die nachfolgend dargestellten Medien die von Ihnen geschätzte Nutzungsdauer für Ihren Physikunterricht an. Stellen Sie sich dabei eine typische, von Ihnen geplante Unterrichtseinheit in der Sekundarstufe I, z. B. zum Thema Elektrizitätslehre, vor. Schätzen Sie ab, wie lange Sie in Ihrem Physikunterricht das jeweilige Medium nutzen, wenn Sie ein Inhaltsgebiet über vier Wochen hinweg unterrichten (zwei Unterrichtsstunden pro Woche, 360 Minuten in vier Wochen).
      *Bitte geben Sie die geschätzte Zeit in Minuten in der folgenden Tabelle an. Sie haben die Möglichkeit, weitere Medien zu ergänzen.*

| Von den ca. 360 Minuten einer durchschnittlichen Unterrichtseinheit nutzen die Schülerinnen und Schüler … | |
| --- | --- |
| ein gegenständliches Modell (z. B. Wassermodell für den Stromkreis oder die Wellenwanne) | ca. _____ Minuten. |
| eine virtuelle Lernumgebung | ca. _____ Minuten. |
| ein reales Experiment | ca. _____ Minuten. |
| ein Smartphone | ca. _____ Minuten. |
| einen Tablet-PC | ca. _____ Minuten. |
| ein Notebook | ca. _____ Minuten. |
| einen Computer / Desktop PC | ca. _____ Minuten. |
| Cassy, Cobra o. ä. | ca. _____ Minuten. |
| einen graphischen Taschenrechner (mit Sensoren) | ca. _____ Minuten. |
| einen Overhead-Projektor | ca. _____ Minuten. |
| eine interaktive Tafel | ca. _____ Minuten. |
| ein Schulbuch | ca. _____ Minuten. |
| _________________________ | ca. _____ Minuten. |
| _________________________ | ca. _____ Minuten. |

- 1. Haben Sie in den letzten 5 Jahren in der Sekundarstufe I das Thema Elektrizitätslehre unterrichtet?
     □ Ja □ Nein

1.
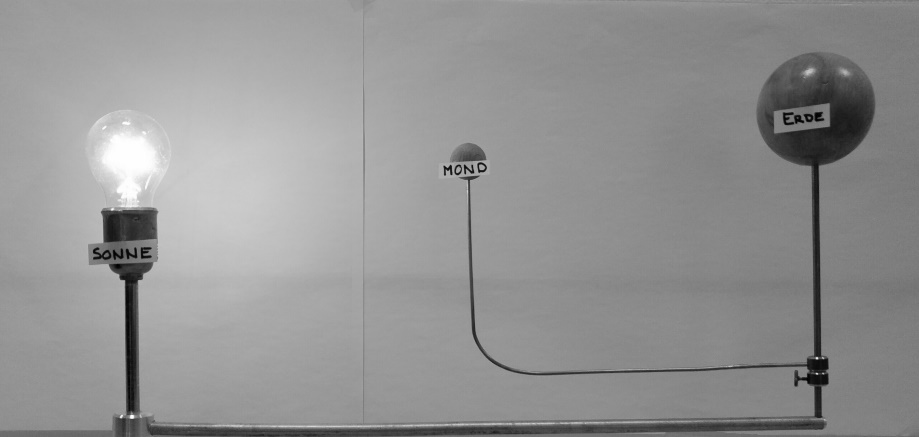
Modelle und Modellexperimente im Physikunterricht

Abbildung 1: Modellexperiment zu den Mondphasen

In der Physik gibt es eine Reihe von Modellexperimenten. Abbildung 1 zeigt den Aufbau eines solchen Modellexperiments. Anhand dieses Modellexperiments können die Mondphasen erklärt werden. Schülerinnen und Schüler können in diesem Zusammenhang eine Kugel als Mond um eine weitere Kugel (Erde) kreisen. Auf der „Erde“ stehen Spielfiguren aus deren Blickwinkel sich die Mondphasen ergeben. Die Lernenden stellen einen Bezug zwischen der Position der Lichtquelle und der Kugeln zueinander sowie dem optischen Eindruck her.

- 1. Modellexperimente für den Physikunterricht sind an meiner Schule …

| □ für alle Lernenden immer verfügbar. |
| --- |
| □ für alle Lernenden bei Bedarf als mobiler Klassensatz verfügbar. |
| □ für alle Lernenden bei Bedarf im Physikraum verfügbar. |
| □ in Form einzelner Beispiele bei Bedarf verfügbar. |
| □ nicht verfügbar. |

Wenn an Ihrer Schule **keine Modelle und Modellexperimente** für den Physikunterricht vorhanden sind, fahren Sie bitte mit Abschnitt C (Virtuelle Lernumgebungen im Physikunterricht) auf S. 9 fort.

- 1. Wie oft führen Ihre Schülerinnen und Schüler folgende Lernaktivitäten bei der Nutzung von Modellen im Physikunterricht typischerweise durch? Denken Sie dabei an **eine konkrete** Klasse, die Sie in Physik unterrichten.

| Meine Schülerinnen und Schüler nutzen Modelle oder Modellexperimente im Physikunterricht … | in keiner oder fast keiner Unterrichtsstunde | in weniger als der Hälfte der Unterrichtsstunden | in mindestens der Hälfte der Unterrichtsstunden | in jeder oder fast jeder Unterrichtsstunde |
| --- | --- | --- | --- | --- |
| zur Beschreibung oder Deutung von Aspekten des Basiskonzepts Energie (Erhaltung, Umwandlung, …). | □ | □ | □ | □ |
| zur Beschreibung der Struktur der Materie oder des Aufbaus von Atomen. | □ | □ | □ | □ |
| zur Deutung von Wechselwirkungen mithilfe des Modells. | □ | □ | □ | □ |
| zum Nachvollziehen eines Systems. | □ | □ | □ | □ |
| zum Durchführen von qualitativen und quantitativen Untersuchungen und zum Überprüfen von Hypothesen. | □ | □ | □ | □ |
| zum Erheben von Daten. | □ | □ | □ | □ |
| zum Herausfinden von Regeln und Zusammenhängen. | □ | □ | □ | □ |
| zum Analysieren von Strukturen und Beziehungen in Daten. | □ | □ | □ | □ |
| zur Beantwortung physikalischer Fragestellungen. | □ | □ | □ | □ |
| zum Veranschaulichen und Erklären physikalischer Sachverhalte. | □ | □ | □ | □ |
| zum Reflektieren, in welchem Verhältnis Modell und Wirklichkeit zueinander stehen. | □ | □ | □ | □ |
| zum Bewerten der Grenzen des Modells. | □ | □ | □ | □ |
|  |  |  |  |  |
| *Hier haben Sie die Möglichkeit weitere Aktivitäten zu ergänzen:* |  |  |  |  |
|  | □ | □ | □ | □ |
|  | □ | □ | □ | □ |

- 1. Alles in allem: Wie zufrieden sind Sie mit der derzeitigen Ausstattung mit gegenständlichen Modellexperimenten für den Physikunterricht an Ihrer Schule?

| sehr unzufrieden | eher unzufrieden | eher zufrieden | sehr zufrieden |
| --- | --- | --- | --- |
| □ | □ | □ | □ |

1. Virtuelle Lernumgebungen im Physikunterricht

*
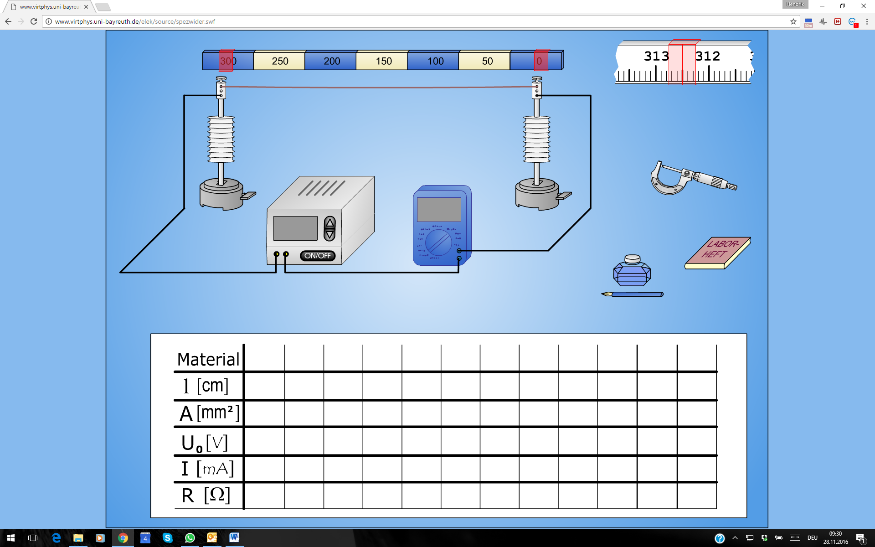
*Durch die Nutzung neuer Medien lassen sich viele Experimente aus dem Physikunterricht auch in virtueller Form durchführen. Neben anderen Beispielen bietet die Homepage der Universität Colorado viele solche Experimente an (s. Abbildung 2). In einem virtuellen Sammlungsraum stehen verschiedene Geräte zum Experimentieren zur Verfügung.

*Abbildung 2: Beispiel für eine virtuelle Lernumgebung. Quelle: http://virtphys.uni-bayreuth.de/elek/*

- 1. Virtuelle Lernumgebungen können im Physikunterricht an meiner Schule …

| □ durch alle Lernenden immer genutzt werden. |
| --- |
| □ durch alle Lernenden bei Bedarf im Klassensatz genutzt werden. |
| □ durch alle Lernenden bei Bedarf im Computerraum genutzt werden. |
| □ durch einzelne Lernende bei Bedarf in Form einzelner Lizenzen genutzt werden. |
| □ nicht genutzt werden. |

Wenn an Ihrer Schule **keine virtuellen Lernumgebungen** für den Physikunterricht vorhanden sind, fahren Sie bitte mit Abschnitt D (Bekanntheitsgrad der Mediennutzung) auf S. 10 fort.

- 1. Wie oft führen Ihre Schülerinnen und Schüler folgende Lernaktivitäten bei der Nutzung von virtuellen Lernumgebungen im Physikunterricht typischerweise durch? Denken Sie dabei an **eine konkrete** Klasse, die Sie in Physik unterrichten.

| Meine Schülerinnen und Schüler nutzen virtuelle Lernumgebungen im Physikunterricht … | in keiner oder fast keiner Unterrichtsstunde | in weniger als der Hälfte der Unterrichtsstunden | in mindestens der Hälfte der Unterrichtsstunden | in jeder oder fast jeder Unterrichtsstunde |
| --- | --- | --- | --- | --- |
| zur Beschreibung oder Deutung von Aspekten des Basiskonzepts Energie (Erhaltung, Umwandlung, …) | □ | □ | □ | □ |
| zur Beschreibung der Struktur der Materie oder des Aufbaus von Atomen. | □ | □ | □ | □ |
| zur Deutung von Wechselwirkungen mithilfe des Modells. | □ | □ | □ | □ |
| um ein System nachvollziehen zu können. | □ | □ | □ | □ |
| zum Entwickeln von Fragestellungen. | □ | □ | □ | □ |
| zum Durchführen von qualitativen und quantitativen Untersuchungen und zum Überprüfen von Hypothesen. | □ | □ | □ | □ |
| zum Erheben von Daten. | □ | □ | □ | □ |
| zum Herausfinden von Regeln und Zusammenhängen. | □ | □ | □ | □ |
| zum Lernen der Bedienung der virtuellen Lernumgebung | □ | □ | □ | □ |
| zum Analysieren von Strukturen und Beziehungen in Daten. | □ | □ | □ | □ |
| zur Beantwortung physikalischer Fragestellungen. | □ | □ | □ | □ |
| zum Veranschaulichen und Erklären physikalischer Sachverhalte. | □ | □ | □ | □ |
| zum Protokollieren des Verlaufs und der Ergebnisse von Untersuchungen und Diskussionen. | □ | □ | □ | □ |
|  |  |  |  |  |
| *Hier haben Sie die Möglichkeit weitere Aktivitäten zu ergänzen:* |  |  |  |  |
|  | □ | □ | □ | □ |
|  | □ | □ | □ | □ |

- 1. Alles in allem: Wie zufrieden sind Sie mit der derzeitigen Ausstattung mit Lizenzen für virtuelle Lernumgebungen für den Physikunterricht an Ihrer Schule?

| sehr unzufrieden | eher unzufrieden | eher zufrieden | sehr zufrieden |
| --- | --- | --- | --- |
| □ | □ | □ | □ |

1. Bekanntheitsgrad der Mediennutzung

Gleich geschafft! Nach dem fachlichen Teil haben wir noch ein paar Fragen, um besser einschätzen zu können, welche Bedürfnisse Sie als Lehrkraft in Bezug auf mediengestütztes Unterrichten haben.

Im Folgenden finden Sie verschiedene Einstellungen, Haltungen und Positionen, die Sie als Lehrkraft während der gedanklichen Beschäftigung mit dem mediengestützten Unterrichten bzw. während der Umsetzung mediengestützten Unterrichtens haben können. Es ist möglich, dass Sie bereits mit dem mediengestützten Unterrichten mit Modellexperimenten und/oder virtuellen Lernumgebungen vertraut sind oder dass Ihnen dieses Unterrichten wenig geläufig ist. Es kann auch sein, dass die Inhalte mancher Aussagen für Sie zurzeit gar keine Relevanz besitzen. Gehen Sie daher bei der Beantwortung folgendermaßen vor:

Kreuzen Sie bitte an, wie stark die Aussage auf **Sie persönlich** zum **jetzigen Zeitpunkt** zutrifft. Hierfür stehen Ihnen sieben Abstufungen (1 bis 7) zur Verfügung. Wenn Sie aber gar nichts mit der Aussage anfangen können (z. B. weil Sie sich noch nie Gedanken dazu gemacht haben), dann kreuzen Sie bitte das Kästchen „zurzeit nicht relevant“ (0) an. Bitte beantworten Sie die folgenden Aussagen nach der eben beschriebenen Vorgehensweise.

|  | Medium: Modelle und Modellexperimente | | | | | | | |  | Medium: virtuelle Lernumgebungen | | | | | | | |
| --- | --- | --- | --- | --- | --- | --- | --- | --- | --- | --- | --- | --- | --- | --- | --- | --- | --- |
|  | 0 zurzeit nicht relevant | 1 trifft zurzeit gar nicht auf mich zu | 2 | 3 | 4 | 5 | 6 | 7 trifft zurzeit völlig auf mich zu |  | 0 zurzeit nicht relevant | 1 trifft zurzeit gar nicht auf mich zu | 2 | 3 | 4 | 5 | 6 | 7 trifft zurzeit völlig auf mich zu |
| Ich würde mich gerne darüber austauschen, wie man mathematisch-naturwissenschaftlichen Unterricht mediengestützt gestalten kann. | □ | □ | □ | □ | □ | □ | □ | □ |  | □ | □ | □ | □ | □ | □ | □ | □ |
| Ich wüsste gerne, welche Ressourcen dafür zur Verfügung stehen. | □ | □ | □ | □ | □ | □ | □ | □ |  | □ | □ | □ | □ | □ | □ | □ | □ |
| Ich will wissen, inwiefern das Unterrichten mit dem Medium besser ist. | □ | □ | □ | □ | □ | □ | □ | □ |  | □ | □ | □ | □ | □ | □ | □ | □ |
| Ich würde gerne wissen, wie sich meine Aufgaben beim Unterrichten mit Medien konkret verändern sollen. | □ | □ | □ | □ | □ | □ | □ | □ |  | □ | □ | □ | □ | □ | □ | □ | □ |
| Ich hätte gerne mehr Informationen über den nötigen Arbeits- und Zeitaufwand beim Unterrichten mit Medien. | □ | □ | □ | □ | □ | □ | □ | □ |  | □ | □ | □ | □ | □ | □ | □ | □ |
| Ich möchte gerne wissen, wie sich meine Rolle durch den Einsatz des Mediums verändert. | □ | □ | □ | □ | □ | □ | □ | □ |  | □ | □ | □ | □ | □ | □ | □ | □ |
| Ich mache mir Gedanken über die Auswirkungen von mediengestütztem Unterrichten auf die Schülerinnen und Schüler. | □ | □ | □ | □ | □ | □ | □ | □ |  | □ | □ | □ | □ | □ | □ | □ | □ |
| Ich überlege wie ich die Wirkung der Medien auf die Schülerinnen und Schüler überprüfen kann. | □ | □ | □ | □ | □ | □ | □ | □ |  | □ | □ | □ | □ | □ | □ | □ | □ |
| Ich möchte Schülerrückmeldungen dazu nutzen, um die Verwendung von Medien fortzuentwickeln. | □ | □ | □ | □ | □ | □ | □ | □ |  | □ | □ | □ | □ | □ | □ | □ | □ |
| Ich würde gerne mit anderen Lehrkräften zum unterrichtspraktischen Einsatz von Medien zusammenarbeiten. | □ | □ | □ | □ | □ | □ | □ | □ |  | □ | □ | □ | □ | □ | □ | □ | □ |
| Ich möchte gerne meine Aktivitäten mit anderen koordinieren, um die positive Wirkung des Medieneinsatzes im Unterricht zu maximieren. | □ | □ | □ | □ | □ | □ | □ | □ |  | □ | □ | □ | □ | □ | □ | □ | □ |
| Mich interessiert, was andere Lehrkräfte auf dem Gebiet des mediengestützten Unterrichts unternehmen. | □ | □ | □ | □ | □ | □ | □ | □ |  | □ | □ | □ | □ | □ | □ | □ | □ |

1. Tablets oder Taschenrechner mit Sensoren im Physikunterricht

Für die Durchführung von Physikunterricht wird aktuell die Nutzung von digitalen Medien diskutiert. Zunehmend stehen in Schulen digitale Medien wie Tablets oder Taschenrechner mit Sensoren zur Verfügung.

- 1. Bitte legen Sie zunächst fest, welches der beiden Systeme Sie prinzipiell häufiger im Physikunterricht verwenden:

| □ Tablets oder Smartphones |
| --- |
| □ Taschenrechner mit Sensoren |
| □ Keines von Beidem |

- 1. Bitte geben Sie für das Medium an, welches Sie gerade ausgewählt haben an:

Die oben gewählten Endgeräte sind an meiner Schule zur Nutzung im Physikunterricht ...

| □ | für alle Lernenden immer verfügbar. |
| --- | --- |
| □ | für alle Lernenden bei Bedarf als mobiler Klassensatz verfügbar. |
| □ | für alle Lernenden bei Bedarf in einem Fachraum, z. B. Computerraum oder Physikraum, verfügbar. |
| □ | in Form einzelner Geräte bei Bedarf verfügbar. |
| □ | nicht verfügbar. |

- 1. Wie oft führen Ihre Schülerinnen und Schüler die nachfolgend aufgeführten Lernaktivitäten im Physikunterricht mit dem Medium durch, dass Sie gerade ausgewählt haben? Denken Sie dabei an **eine konkrete** Klasse, die Sie in Physik unterrichten.

| Meine Schülerinnen und Schüler nutzen die Endgeräte im Physikunterricht … | in keiner oder fast keiner Unterrichtsstunde | in weniger als der Hälfte der Unterrichtsstunden | in mindestens der Hälfte der Unterrichtsstunden | in jeder oder fast jeder Unterrichtsstunde |
| --- | --- | --- | --- | --- |
| zum Erstellen von Präsentationen bzw. Präsentieren von Ergebnissen. | □ | □ | □ | □ |
| zum Durchführen einer Rechnung. | □ | □ | □ | □ |
| zum Recherchieren von Informationen (falls technisch möglich). | □ | □ | □ | □ |
| zum Erstellen von Tabellen oder Diagrammen in der Auswertung eines Experiments. | □ | □ | □ | □ |
| zum Kommunizieren mit Mitschülerinnen und Mitschülern. | □ | □ | □ | □ |
| zum Überprüfen des aktuellen Leistungsstandes. | □ | □ | □ | □ |
| zum Simulieren physikalischer oder technischer Prozesse. | □ | □ | □ | □ |
| zum Messen von Beschleunigung, Temperatur, o .ä. | □ | □ | □ | □ |
| zum Bearbeiten von Filmen und Bildern, z. B. Videoanalyse von Bewegungen (nur bei Smartphones oder Tablets). | □ | □ | □ | □ |
|  |  |  |  |  |
| *Hier haben Sie die Möglichkeit weitere Aktivitäten zu ergänzen:* |  |  |  |  |
|  | □ | □ | □ | □ |
|  | □ | □ | □ | □ |

- 1. Alles in allem: Wie zufrieden sind Sie mit der derzeitigen Ausstattung der oben gewählten Endgeräte?

| sehr unzufrieden | eher unzufrieden | eher zufrieden | sehr zufrieden |
| --- | --- | --- | --- |
| □ | □ | □ | □ |

1. Einstellungen zum Medieneinsatz allgemein
   1. Im Folgenden finden Sie verschiedene Aussagen zu digitalen Medien im Unterricht. Geben Sie bitte an, inwiefern Sie den Aussagen zustimmen. Es gibt dabei keine richtigen oder falschen Antworten. Bitte setzen Sie je Aussage nur ein Kreuz, das auf Ihren Standpunkt am besten zutrifft.

|  | stimme überhaupt nicht zu | stimme eher nicht zu | neutral | stimme eher zu | stimme voll zu |
| --- | --- | --- | --- | --- | --- |
| Digitale Medien im Unterricht haben nur Unterhaltungswert für Schülerinnen und Schüler. | □ | □ | □ | □ | □ |
| Digitale Medien können den Lernerfolg bei Schülerinnen und Schülern positiv beeinflussen. | □ | □ | □ | □ | □ |
| Digitale Medien verschlechtern die Qualität des Unterrichts. | □ | □ | □ | □ | □ |
| Digitale Medien unterstützen Schülerinnen und Schüler beim Lernen. | □ | □ | □ | □ | □ |
| Digitale Medien sorgen im Unterricht für Unruhe und lenken vom Lerninhalt ab. | □ | □ | □ | □ | □ |
| Der Einsatz von digitalen Medien macht den Unterricht effektiver und effizienter. | □ | □ | □ | □ | □ |

- 1. Wie würden Sie Ihr Verhältnis zu digitalen Medien generell beschreiben?

| Ich kann mit elektronischen Medien nichts anfangen | Ich stehe elektronischen Medien eher skeptisch gegenüber | Ich stehe elektronischen Medien eher positiv gegenüber | Ich bin ein Technik-Fan |
| --- | --- | --- | --- |
| □ | □ | □ | □ |

- 1. Wie stehen Sie dem Einsatz digitaler Medien im Unterricht im Großen und Ganzen gegenüber?

| negativ | eher negativ | eher positiv | positiv |
| --- | --- | --- | --- |
| □ | □ | □ | □ |

- 1. Bitte bewerten Sie, inwiefern Sie den folgenden Aussagen zustimmen.

|  | stimmt nicht | stimmt kaum | stimmt eher | stimmt genau |
| --- | --- | --- | --- | --- |
| Ich kann in meiner Unterrichtsplanung zu den Lernzielen passende Ein­sätze digitaler Medien planen, auch wenn meine Schule nicht optimal mit digitalen Medien ausgestattet ist. | □ | □ | □ | □ |
| Ich kann den Einsatz digitaler Medien im Fachunterricht so planen, dass meine Schülerinnen und Schüler begeistert sind, auch wenn sie sich sonst wenig für das Fach interessieren. | □ | □ | □ | □ |
| Ich kann den Einsatz eines digitalen Mediums im Fachunterricht didaktisch begründen, auch wenn ich dieses digitale Medium noch nicht selbst eingesetzt habe. | □ | □ | □ | □ |
| Ich kann eine Lösung für technische Probleme beim Unterrichten mit digitalen Medien finden, auch wenn ich unter Zeitdruck stehe. | □ | □ | □ | □ |
| Ich kann den Einsatz digitaler Medien so gestalten, dass meine Schülerinnen und Schüler motiviert sind mitzuarbeiten, auch wenn es sich um eine unbeliebte Randstunde handelt. | □ | □ | □ | □ |
| Ich kann den fachlichen Lernprozess durch den Einsatz digitaler Medien unterstützen, auch wenn unvorhergesehene Verständnisschwierigkeiten auftreten. | □ | □ | □ | □ |

- 1. Was ist Ihrer Meinung nach die größte Herausforderung für den Unterricht mit digitalen Medien?

|  |
| --- |

- 1. Haben Sie Anmerkungen zum Fragebogen? Geben Sie uns Feedback, damit wir den Fragebogen besser gestalten können!

|  |
| --- |

**Vielen Dank für Ihre Unterstützung!**
