# Supplementary material for: Is There a Subject Specific Use of Media in Science? Results of a Questionnaire Survey
Source: Z Didakt Nat Wiss. 2021 Jun 18;27(1):139–54. [Article in German] doi: 10.1007/s40573-021-00130-5 (PMC8212071; doi:10.1007/s40573-021-00130-5)
Supplement: Supplementary file 4 [file 40573_2021_130_MOESM4_ESM.docx]

# Skala „Selbstwirksamkeitserwartung beim Unterrichten mit digitalen Werkzeugen“

### Verwendete Skala

Bitte bewerten Sie, inwiefern Sie den folgenden Aussagen zustimmen.

|  |  | stimmt nicht | stimmt kaum | stimmt eher | stimmt genau |
| --- | --- | --- | --- | --- | --- |
| *digM1* | Ich kann in meiner Unterrichtsplanung zu den Lernzielen passende Ein­sätze digitaler Medien planen, auch wenn meine Schule nicht optimal mit digitalen Medien ausgestattet ist. | □ | □ | □ | □ |
| *digM2* | Ich kann den Einsatz digitaler Medien im Fachunterricht so planen, dass meine Schülerinnen und Schüler begeistert sind, auch wenn sie sich sonst wenig für das Fach interessieren. | □ | □ | □ | □ |
| *digM3* | Ich kann den Einsatz eines digitalen Mediums im Fachunterricht didaktisch begründen, auch wenn ich dieses digitale Medium noch nicht selbst eingesetzt habe. | □ | □ | □ | □ |
| *digM4* | Ich kann eine Lösung für technische Probleme beim Unterrichten mit digitalen Medien finden, auch wenn ich unter Zeitdruck stehe. | □ | □ | □ | □ |
| *digM5* | Ich kann den Einsatz digitaler Medien so gestalten, dass meine Schülerinnen und Schüler motiviert sind mitzuarbeiten, auch wenn es sich um eine unbeliebte Randstunde handelt. | □ | □ | □ | □ |
| *digM6* | Ich kann den fachlichen Lernprozess durch den Einsatz digitaler Medien unterstützen, auch wenn unvorhergesehene Verständnisschwierigkeiten auftreten. | □ | □ | □ | □ |

### Konzeption der Skala

|  | Entsprechung nach Meinhardt, Rabe & Krey (2016). Selbstwirksamkeitserwartungen in physikdidaktischen Handlungsfeldern, Handlungsfeld Experimentieren (SWE-EX), Dimensionen Planung (exp) und Durchführung (exd) | |
| --- | --- | --- |
| *digM1* | *exp1* | Ich kann in meiner Unterrichtsplanung zu den Lernzielen passende Experimente aufbauen, auch wenn die Physiksammlung schlecht ausgestattet ist. |
| *digM2* | *exp6* | Ich kann ein Experiment planen, das meine Schülerinnen und Schüler begeistert, auch wenn sie sich sonst wenig für Physik interessieren. |
| *digM3* | *Keine direkte Entsprechung, enthält den Aspekt der didaktischen Begründung ohne hohe Vertrautheit wie in exp7* | |
| *digM4* | *exd5* | Ich kann ein Experiment, das im Physikunterricht nicht auf Anhieb funktioniert, zum Laufen bringen, auch wenn ich unter Zeitdruck stehe. |
| *digM5* | *exd8* | Ich kann ein Experiment so inszenieren, dass meine Schülerinnen und Schüler motiviert sind mitzuarbeiten, auch wenn es sich um eine unbeliebte Randstunde handelt. |
| *digM6* | *Keine direkte Entsprechung, enthält Aspekte von exd6, exd7 und exd9* | |

Literatur:

Meinhardt, C., Rabe, T. &Krey, O. (2016). Selbstwirksamkeitserwartung in physikdidaktischen Handlungsfeldern. Skalendokumentation. Version 1. Online verfügbar unter <https://www.pedocs.de/volltexte/2016/11818/additional/Meinhardt_2016_Selbstwirksamkeitserwartungen_komprimiert.pdf> [28.03.2019]
